# Histocompatibility Complex Status and Mendelian Randomization Analysis in Unsolved Antibody Deficiency

**DOI:** 10.3389/fimmu.2020.00014

**Published:** 2020-01-24

**Authors:** Hassan Abolhassani, Che Kang Lim, Asghar Aghamohammadi, Lennart Hammarström

**Affiliations:** ^1^Division of Clinical Immunology, Department of Laboratory Medicine, Karolinska Institutet at Karolinska University Hospital Huddinge, Stockholm, Sweden; ^2^Research Center for Immunodeficiencies, Pediatrics Center of Excellence, Children's Medical Center, Tehran University of Medical Sciences, Tehran, Iran

**Keywords:** primary immunodeficiency, antibody deficiency, common variable immunodeficiency (CVID), whole exome sequencing, full-resolution MHC typing, Mendelian randomization

## Abstract

The pathogenesis in the majority of patients with common variable immunodeficiency (CVID), the most common symptomatic primary immunodeficiency, remains unknown. We aimed to compare the minor and major histocompatibility complex (MHC) markers as well as polygenic scores of common genetic variants between patients with monogenic CVID and without known genetic mutation detected. Monogenic patients were identified in a CVID cohort using whole exome sequencing. Computational full-resolution MHC typing and confirmatory PCR amplicon-based high-resolution typing were performed. Exome-wide polygenic scores were developed using significantly different variants and multi-variant Mendelian randomization (MR) analyses were used to test the causality of significant genetic variants on antibody levels and susceptibility to infectious diseases. Among 83 CVID patients (44.5% females), monogenic defects were found in 40 individuals. Evaluation of the remaining CVID patients without known genetic mutation detected showed 13 and 27 significantly associated MHC-class I and II alleles, respectively. The most significant partial haplotype linked with the unsolved CVID was W^*^01:01:01-DMA^*^01:01:01-DMB^*^01:03:01:02-TAP1^*^01:01:01 (*P* < 0.001), where carriers had a late onset of the disease, only infection clinical phenotype, a non-familial form of CVID, post-germinal center defects and a non-progressive form of their disease. Exclusion of monogenic diseases allowed MR analyses to identify significant genetic variants associated with bacterial infections and improved discrepancies observed in MR analyses of previous GWAS studies with low pleiotropy mainly for a lower respiratory infection, bacterial infection and Streptococcal infection. This is the first study on the full-resolution of minor and major MHC typing and polygenic scores on CVID patients and showed that exclusion of monogenic forms of the disease unraveled an independent role of MHC genes and common genetic variants in the pathogenesis of CVID.

## Introduction

Common variable immunodeficiency (CVID) is the most common symptomatic primary immunodeficiency (PID), characterized by impairment of antibody production, recurrent infections and immune dysregulation, in particular, autoimmunity (1). Several different pathogeneses have been suggested from which monogenic diseases cover between 10 and 20% (in Western cohorts) up to 68% (in countries with a high rate of consanguinity) (2–4). Approximately 400 genes have been identified as causative defects of PID, from which half of them have been linked to impaired antibody production and CVID (2). However, additional genetic and non-genetic models have also been considered for CVID (5–7).

Minor and major histocompatibility complex (MHC) genes are the most polymorphic genomic region and specific MHC loci determine the presentation of antigens via B cells to T cells to elicit a germinal center reaction. Physiologically, both MHC class I and II molecules are critical in B cells for stimulating antibody class switching and affinity maturation (MHC class II primarily to follicular helper T cells) and supporting presentation of polysaccharides antigens (mainly via positive signals of MHC class I primarily to natural killer T-cells) (8–10). Given the high prevalence of autoimmune disorders in CVID patients, several studies have investigated the frequency of different MHC alleles in subgroups of CVID patients (11, 12). Furthermore, few reports from different ethnic CVID cohorts have also emphasized a possible contribution of the MHC, mainly class II molecules in patients with gastrointestinal autoimmunity, on the predisposition to CVID (13). In some multiplex families with co-occurrence of CVID and selective immunoglobulin A (IgA), deficiency, MHC markers have also been suggested to play a role both in inheritance and as predictors of progressive disease (14).

However, in CVID patients with a lack of identified monogenic mutations, the disease may occur due to polygenic inheritance involving many common genetic variants with a small effect. An improved methodology for calculating polygenic scores, using a larger cohort of sequenced samples and advanced algorithms, has been proposed to identify a combined impact of single nucleotide polymorphisms (SNP) that significantly increase the risk of disease (15). Mendelian randomization (MR) is an analytical method for identification of causality using polygenic variables and it has been successfully implemented for diseases conferring both monogenic and polygenic traits (16).

Hence we compared the MHC markers and polygenic predictors of CVID patients without a molecular genetic diagnosis after whole exome sequencing (WES), where computational analysis based on high-resolution MHC typing from WES data was performed for the first time. Multi-SNP MR analyses using summary-level data from WES were performed to cross-validate the causality of currently identified variants and previously suggested SNPs for antibody deficiency.

## Materials and Methods

### Study Design and Participants

Patients with a diagnosis of CVID based on the updated clinical diagnostic criteria of the European Society for Immunodeficiencies (ESID, https://esid.org/Working-Parties/Registry-Working-Party/Diagnosis-criteria) and the American Academy of Allergy, Asthma & Immunology (AAAAI) practice parameter for the diagnosis and management of PID (17), were recruited from a cohort of antibody deficiency patients evaluated by WES (2). This cohort was designed to investigate the contribution of genetic, immunologic, and clinical factors of the disease.

Among all registered CVID patients in the Iranian national PID registry (18, 19), available individuals who were referred to the Children's Medical Center (Pediatrics Center of Excellence affiliated to Tehran University of Medical Sciences, Tehran, Iran) and completed the molecular diagnostic investigation were consecutively recruited into this study. Written informed consent for the performed evaluations was obtained from all patients and/or their parents, according to the principles of the ethics committee of the Tehran University of Medical Sciences. An evaluation document was used to summarize the demographic information of the patients, including gender, date of birth, clinical parameters and previous medical history, family history, laboratory and molecular data. A computerized database program (new registry section at, http://rcid.tums.ac.ir/) was designed for the final data collection and direct generation for statistical analysis of data.

### Systematic Phenotyping and Genotyping

All clinically diagnosed CVID patients were re-evaluated for fulfilling either the probable or possible diagnostic criteria, and secondary causes of antibody deficiency were ruled out. Clinical phenotyping was performed using a standard method of phenotype subdivision which has been shown to correlate with the quality of life and morbidity among patients with infections only, autoimmunity, lymphoproliferation and enteropathy (20). Based on the epidemiologic data of CVID cohorts worldwide, there are two peak ages of onset, one before the age of ten and another in the third decade of life. Therefore, we defined the early onset as disease onset before age 10 years (20).

Complete blood count, lymphocyte subpopulations, serum Ig levels, and specific antibody responses were measured as previously described (2). Immunological tests were repeated for each patient every 6-months within during routine follow-up visits after the time of diagnosis to evaluate the progression of their antibody deficiency. Patients were classified immunologically based on the main classification for B-cell subsets known as B-cell pattern classification with relevance to genetic findings (21).

Regarding genetic diagnosis, WES was performed according to the protocol described previously (22). For analysis of WES, we followed a published pipeline for prioritizing candidate variants, predicting their effect on protein, homozygosity mapping, large deletion and copy number variation (CNV) detection (2, 22). To classify a patient as a monogenic disease, the pathogenicity of the disease attributable gene variant was re-evaluated using the updated guideline for interpretation of molecular sequencing by the American College of Medical Genetics and Genomics (ACMG), considering the allele frequency in the relevant population database, computational data, immunological/multiomics functional data, familial segregation, parental data and clinical phenotyping (23). In the remaining patients not only CVID associated genes, but also all known other PID genes were normal (2), therefore we labeled them as “without known genetic mutation detected or unsolved” even though there is a possibility of yet unknown inherited disorder in a minority of them.

### MHC Typing Algorithm and Confirmation

The WES data were used for MHC typing of both monogenic and unsolved CVID patients using the major module of Optitype (24) [>97% accuracy (25)]. This algorithm was run according to instructions using fastq files after filtering low-quality reads (base quality of <20 for more than 80% of bases) as an input. In brief, the input data were mapped to the hg38 human reference assembly, and relevant MHC reads from the Binary Alignment Map (BAM) file (chromosome 6, position 29,886,751–33,090,696) were filtered according to their quality scores. Subsequently, four-digit typing, zygosity status and full-resolution imputation were computed. Multiple predictions for an allele at a locus detected in MHC reporter were considered as ambiguous results, and only the first field information was used (26). Confirmatory PCR amplicon-based high-resolution typing was performed on the genomic DNA of the patients as described previously (14, 27).

### Polygenic Model and Mendelian Randomization

Exome-wide association study has previously been conducted on 535,486 SNPs extracted from high-throughput sequencing data of both monogenic and unsolved CVID patients as well as 141,456 individuals at Genome Aggregation Database (gnomAD) and 2,497 individuals at Greater Middle East (GME) Variome Project. All SNPs were mapped to build relevant coordinates using liftOver. Prior to imputation, we removed variants with a genotyping rate <98%, ambiguitious SNPs, evidence of deviation from Hardy-Weinberg equilibrium in controls (*p* < 1 × 10^−4^), and minor allele frequency < 1 × 10^−6^. We conducted χ^2^ tests of association on genotypes for each cohort separately, using only variants that overlapped between patients cohort and controls. We subsequently only included in the analysis the near-independent SNPs that do not account for linkage disequilibrium (LD) and were significantly different between monogenic and unsolved patients for ease of directly comparing the results.

MR analysis was performed using the identified significant genetic variants, in order to evaluate the effect of exclusion of monogenic patients for prediction of independent common variants without confounding factors, as instrumental variables (serum Ig level) to test for causality (bacterial infections). The result of the MR model on current predictor SNPs of unsolved CVID patients was empowered by comparison of multiple genetic variants reported on previously independent studies on antibody levels using the genome-wide association (GWAS) catalog provided by the National Human Genome Research Institute (NHGRI) and the European Bioinformatics Institute (EMBL-EBI, https://www.ebi.ac.uk/gwas/). Selection of GWAS catalogs on the infectious outcomes were performed to test the causality influenced by the exposures, including ICD10 codes of: J22 Unspecified acute lower respiratory infection (UKB-a:540, *n* = 337,199 individuals), A49.9 Bacterial infections of unspecified site (UKB-b:1605, *n* = 463,010), A49.8 Other bacterial infections of unspecified site (UKB-b:1399, *n* = 463,010), A49.0 Staphylococcal infection, unspecified (UKB-b:3266, *n* = 463,010), 0410 Streptococcus infection (UKB-b:4251, *n* = 463,010) and A49.1 Streptococcal infection, unspecified (UKB-b:4884, *n* = 463,010). Recruitment of GWAS catalogs were performed in the MR-base analytical platform established by the MRC Integrative Epidemiology Unit (University of Bristol, http://app.mrbase.org).

### Statistical Approach

Statistical analysis was performed using SPSS (version 21.0.0, SPSS, Chicago, Illinois) and R statistical systems (version 3.4.1.; R Foundation for Statistical Computing, Vienna, Austria) software to compare clinical and immunological parameters between patients with an identified genetic defect and patients with no genetic diagnosis. The one-sample Kolmogorov-Smirnov test was applied to estimate whether data distribution was normal. Parametric and non-parametric analyses were performed based on the finding of this evaluation. Regarding MR, we used the proxy SNPs method instead of LD tagging with minimum LD values of 0.8 and minor allele frequency of (MAF) threshold of aligning palindromes as 0.3. Several MR methods with different sensitivities were applied including Wald ratio, MR Egger, weighted median, and inverse variance weighted algorithms. Forest plot and funnel plot were used to illustrate causality effects and horizontal pleiotropy, respectively. A *P*-value of <5 × 10^−8^ was considered for multiple testing and selection of significant SNPs and *P*-value <0.05 was assumed for comparisons of monogenic and unsolved CVID patients as statistically significant.

## Results

Among all genetically evaluated CVID patients, 83 patients agreed to participate in this study (Table 1) and monogenic defects were found and confirmed in 40 individuals (2). The remaining 43 “idiopathic” CVID patients were labeled as an unsolved patient. The studied patients (46 males, 37 females) from 71 unrelated kindreds were mainly children and adolescents at the time of the study (43 patients were <18 years old) and parental consanguinity was recorded in 64 patients. The median age of the patients at the onset of symptoms was 3 years (range 0.5–36 years; early-onset manifestation in 79.5%) and the median diagnostic delay (the gap between the onset of the symptoms and diagnosis of CVID) was 4 years (range 0.4–39 years). Of note, 11 patients were from multiplex families (classified as familial cases, 36% with an unsolved disease) and 7 cases progressed to CVID from another form of antibody deficiency during the course of the disease (IgA deficiency and IgG subclass deficiency, 14.2% with an unsolved disease). A summary of the clinical and immunologic phenotype of the studied patients is provided in Table 1. There was a significant difference among patients with or without monogenic disorders regarding the age of onset and progressive form of CVID, while parental consanguinity and familial cases were comparable.

**Table 1:** Clinical and immunologic phenotypes of the 83 CVID patients included in the study.

**Parameters**	**Total CVID patients (*n* = 83)**	**Monogenic patients (*n* = 40)**	**Unsolved patients (*n* = 43)**	***P*-value**
Gender (M/F)	46/37	20/20	26/17	0.16
Median current age, year (range)	18 (5–44)	16 (5–26)	21 (6–44)	0.08
Median age of onset, year (range)	2 (0.5–36)	1.0 (0.5–10)	4.5 (3–36)	0.04^*^
Median age of diagnosis, year (range)	8 (0.5–30)	7 (0.5–20)	10 (1–30)	0.09
Parental consanguinity (%)	64 (77.1)	31(77.5)	33(76.7)	0.46
Familial patients (%)	11 (13.2)	7 (17.5)	4 (9.3)	0.13
**CLINICAL PHENOTYPE**				
Infections only (%)	25 (30.1)	9 (22.5)	16 (37.2)	0.07
Autoimmunity (%)	29 (34.9)	16 (40.0)	13 (30.2)	0.17
Lymphoproliferation (%)	30 (36.1)	16 (40.0)	14 (32.5)	0.24
Enteropathy (%)	23 (27.7)	11 (27.5)	12 (27.9)	0.48
Malignancy (%)	4 (4.8)	3 (7.5)	1 (2.3)	0.13
Allergy (%)	10 (12.0)	7 (17.5)	3 (6.9)	0.07
Overlap phenotype (%)^***^	31 (37.3)	18 (45)	13 (30.2)	0.08
**IMMUNOLOGIC PHENOTYPE**				
Progressive form of antibody deficiency (%)	7 (8.4)	6 (15)	1 (2.3)	0.01^*^
White blood cells/ul (SD)	8,051.5 (3,504.2)	7,955.8 (2,119.0)	8,257.2 (2,738.9)	0.43
Lymphocytes/ul (SD)	2,890.5 (1,024.9)	2,784.0 (1,935.4)	3,137.2 (2,344.0)	0.31
B cells, % (SD)	10.5 (7.8)	10.2 (4.4)	11.3 (5.9)	0.49
IgM, mg/dl (SD)	18.7 (10.2)	17.3 (7.5)	21.1 (6.8)	0.24
IgG, mg/dl (SD)^**^	275.9 (251.3)	337.8 (229.1)	241.0 (142.8)	0.09
IgA, mg/dl (SD)	22.8 (12.0)	27.8 (11.9)	13.9 (10.6)	0.07
**B CELL SUBSET PHENOTYPE**				
Pattern 1 (low transitional and memory B cells) (%)	23 (27.7)	8 (20.0)	10 (23.2)	0.35
Pattern 2 (low naïve mature, marginal zone-like and memory B cells) (%)	6 (7.2)	3 (7.5)	3 (6.9)	0.46
Pattern 3 (low marginal zone-like and memory B cells) (%)	11 (13.2)	7 (17.5)	4 (9.3)	0.13
Pattern 4 (low memory B cells) (%)	26 (31.3)	15 (37.5)	11 (25.5)	0.12
Pattern 5 (post-germinal center defect) (%)	17 (20.4)	7 (17.5)	15 (34.8)	0.04^*^

* *Statistically significant difference, p < 0.05*.

** *Values at the time of CVID diagnosis and before immunoglobulin substitution*.

*** *Overlap phenotype: CVID patients that develop more than one non-infectious complications and present with at least two concurrent complications of autoimmunity, lymphoproliferation, and enteropathy (2, 20)*.

**Figure 1:**
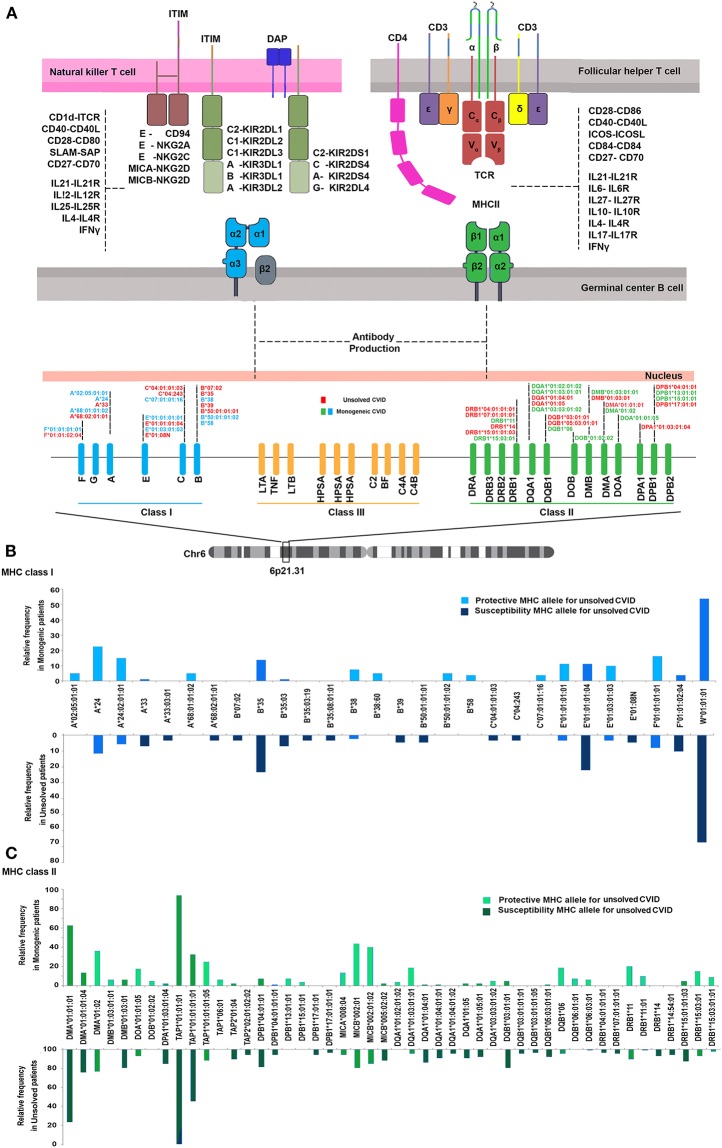
**(A)** Schematic presentation of association of MHC status in the pathogenesis of common variable immunodeficiency based on the role of MHC in antigen presentation within germinal center B cells. **(B)** MHC class I **(C)** and MHC class II risk and protective alleles for unsolved CVID compared to monogenic-CVID patients.

We first investigated the frequency of MHC class I and II alleles in the CVID patients. Among the 83 patients, high-resolution WES-based typing of class I revealed the highest diversity in MHC-B with 84 unique alleles (mainly B^*^35, 31 alleles out of total 166 alleles:18.6%). However, the CVID cohort had a restricted MHC-H repertoire with only 4 unique alleles (mainly H^*^02, 113 alleles: 68.0%, Tables S1–S8). Evaluation of MHC class II showed that the most diverse locus was MHC-DPB1 with 40 unique alleles (mainly DPB1^*^463:01:01, 23 alleles: 13.8%), in contrast to three unique alleles for MHC-DRB4 (mainly DRB4^*^01:03:01, 135 alleles: 81.3%, Tables S9–S23). The most significant increases in the proportions of class I in the unsolved patient cohort were observed in B^*^39 (*p* = 0.02), B^*^50:01:01:01 (*p* = 0.02), and E^*^01:08N (*p* = 0.02, Table 2, Figures 1A,B). Moreover, susceptibility class II regions for unsolved CVID were most significantly associated with DQA1^*^01:04:01 (*p* < 0.001), DQB1^*^03:01:01 (*p* = 0.002), DPA1^*^01:03:01:04 (*p* = 0.002), and TAP1^*^01:01:01:01(*p* = 0.002, Table 3, Figures 1A,C). There were no significant differences in the frequency of alleles of MHC–H, –G –DRB3, and –DRB4 between monogenic and unsolved CVID patients (Tables S6, S7, S20, S21, Figure 1).

**Table 2:** Significantly different MHC-class I alleles associated with monogenic and unsolved CVID patients.

**MHC class I**	**Monogenic patients (*n* = 40/alleles = 80)**	**Homozygous monogenic patients**	**Unsolved patients (*n* = 43/alleles = 86)**	**Homozygous unsolved patients**	**OR**	**Effect on unsolved CVID**	***P*-value**
A^*^02:05:01:01	4	0	0	0	NI	P	0.017^*^
A^*^24	18	2	10	1	1.93	P	0.03^*^
A^*^24:02:01:01	12	0	5	0	2.58	P	0.02^*^
A^*^33	1	0	6	0	0.17	S	0.03^*^
A^*^33:03:01	0	0	3	0	NI	S	0.04^*^
A^*^68:01:01:02	4	0	0	0	NI	P	0.017^*^
A^*^68:02:01:01	0	0	3	0	NI	S	0.04^*^
B^*^07:02	0	0	3	0	NI	S	0.04^*^
B^*^35	11	2	20	4	0.59	S	0.05^*^
B^*^35:03	1	0	6	1	0.17	S	0.03^*^
B^*^35:03:19	0	0	3	0	NI	S	0.04^*^
B^*^35:08:01:01	0	0	3	0	NI	S	0.04^*^
B^*^38	6	0	2	0	3.22	P	0.05^*^
B^*^38:60	4	0	0	0	NI	P	0.017^*^
B^*^39	0	0	4	0	NI	S	0.02^*^
B^*^50:01:01:01	0	0	4	1	NI	S	0.02^*^
B^*^50:01:01:02	4	0	0	0	NI	P	0.017^*^
B^*^58	3	0	0	0	NI	P	0.03^*^
C^*^04:01:01:03	0	0	3	0	NI	S	0.04^*^
C^*^04:243	0	0	3	1	NI	S	0.04^*^
C^*^07:01:01:16	3	1	0	0	NI	P	0.03^*^
E^*^01:01:01:01	9	3	3	0	3.22	P	0.02^*^
E^*^01:01:01:04	9	1	19	5	0.50	S	0.03^*^
E^*^01:03:01:03	8	2	3	1	2.86	P	0.04^*^
E^*^01:08N	0	0	4	1	NI	S	0.02^*^
F^*^01:01:01:01	13	2	7	0	1.99	P	0.05^*^
F^*^01:01:02:04	3	0	9	2	0.35	S	0.04^*^
W^*^01:01:01	43	4	58	2	0.79	S	0.03^*^

*OR, odds ratio; NI, not calculable; P, preventive for unsolved CVID; S, susceptibility for unsolved CVID*.

* *Statistically significant difference, p < 0.05*.

**Table 3:** Significantly different MHC-class II alleles associated with monogenic and non-monogenic CVID patients.

**MHC class II**	**Monogenic patients (*n* = 40/alleles = 80)**	**Homozygous monogenic patients**	**Unsolved patients (*n* = 43/alleles=86)**	**Homozygous unsolved patients**	**OR**	**Effect on unsolved CVID**	***P*-value**
DMA^*^01:01:01	50	21	66	29	0.81	S	0.02^*^
DMA^*^01:01:01:04	11	4	21	8	0.56	S	0.04^*^
DMA^*^01:02	29	10	20	6	1.55	P	0.03^*^
DMB^*^01:03:01:01	5	2	0	0	NI	P	0.009^**^
DMB^*^01:03:01	5	0	17	0	0.31	S	0.005^**^
DOA^*^01:01:05	14	6	6	2	2.50	P	0.01^*^
DOB^*^01:02:02	4	1	0	0	NI	P	0.01^*^
DPA1^*^01:03:01:04	2	0	13	2	0.16	S	0.002^**^
TAP1^*^01:01:01	75	34	86	42	0.94	S	0.009^**^
TAP1^*^01:01:01:01	26	12	47	16	0.60	S	0.002^**^
TAP1^*^01:01:01:05	20	6	10	3	2.15	P	0.01^*^
TAP1^*^06:01	5	1	0	0	NI	P	0.009^**^
TAP2^*^01:04	2	0	9	3	0.23	S	0.01^*^
TAP2^*^02:01:02:02	0	0	5	2	NI	S	0.01^*^
DPB1^*^04:01:01	6	1	16	2	0.40	S	0.01^*^
DPB1^*^04:01:01:01	1	0	5	1	0.21	S	0.03^*^
DPB1^*^13:01:01	6	1	0	0	NI	P	0.004^**^
DPB1^*^15:01:01	3	1	0	0	NI	P	0.03^*^
DPB1^*^17:01:01	0	0	5	0	NI	S	0.01^**^
DPB1^*^17:01:01:01	0	0	3	0	NI	S	0.04^*^
MICA^*^008:04	11	5	5	2	2.36	P	0.04^*^
MICB^*^002:01	35	16	17	6	2.21	P	<0.001^***^
MICB^*^002:01:02	32	15	13	6	2.64	P	<0.001^***^
MICB^*^005:02:02	2	0	10	0	0.21	S	0.01^*^
DQA1^*^01:02:01:02	3	1	0	0	NI	P	0.03^*^
DQA1^*^01:03:01:01	15	6	4	1	4.03	P	0.002^**^
DQA1^*^01:04:01	1	0	12	3	0.08	S	<0.001^***^
DQA1^*^01:04:01:01	1	0	8	2	0.13	S	0.01^*^
DQA1^*^01:04:01:02	0	0	4	1	NI	S	0.02^*^
DQA1^*^01:05	2	0	8	1	0.26	S	0.03^*^
DQA1^*^01:05:01	2	0	7	1	0.30	S	0.05^*^
DQA1^*^03:03:01:02	4	2	0	0	NI	P	0.01^*^
DQB1^*^03:01:01	4	1	17	4	0.25	S	0.002^**^
DQB1^*^03:01:01:01	0	0	4	0	NI	S	0.02^*^
DQB1^*^03:01:01:05	0	0	3	1	NI	S	0.04^*^
DQB1^*^05:03:01:01	1	0	7	0	0.15	S	0.01^*^
DQB1^*^06	15	4	4	0	4.03	P	0.004^**^
DQB1^*^06:01:01	6	3	0	0	NI	P	0.004^**^
DQB1^*^06:03:01	5	1	1	0	5.37	P	0.03^*^
DRB1^*^04:01:01:01	0	0	3	0	NI	S	0.04^*^
DRB1^*^07:01:01:01	0	0	4	0	NI	S	0.02^*^
DRB1^*^11	16	6	9	1	1.91	P	0.04^*^
DRB1^*^11:01	8	3	1	0	8.6	P	0.006^**^
DRB1^*^14	0	0	6	2	NI	S	0.008^**^
DRB1^*^14:54:01	0	0	5	2	NI	S	0.01^**^
DRB1^*^15:01:01:03	4	0	11	0	0.39	S	0.04^*^
DRB1^*^15:03:01	12	4	6	0	2.15	P	0.04^*^
DRB1^*^15:03:01:01	7	3	2	0	3.76	P	0.03^*^

*OR, odds ratio; NI, not calculable; P, preventive for unsolved; CVID, S, susceptibility for unsolved CVID*.

* Statistically significant difference, p < 0.05,

** p < 0.01,

*** *p < 0.001*.

Regression model analysis using the identified significant MHC alleles suggested a combination of B^*^35, DMA^*^01:02, TAP1^*^06:01, MICB^*^002:01, DQA1^*^01:04:01, and DQB1^*^03:01:01 as the best fit model to predict an unsolved form of CVID (*p* = 4.5 × 10^−6^, Table S24). In the second model, we tested for an association with a significant haplotype in patients with unsolved CVID. In this model, the W^*^01:01:01-DMA^*^01:01:01-DMB^*^01:03:01:02-TAP1^*^01:01:01 (*p* < 0.001), was the most significant associated haplotype with an increased unsolved CVID odds (Table 4). This haplotype was exclusively identified in 11 patients without a genetic diagnosis. The majority of these patients had a late onset (*n* = 9, 81.8%) and none of them were of a familial CVID or progressive form of the disease. Infections only phenotype (*n* = 10, 90.9%) and post-germinal center defects (*n* = 8, 72.7%) were the main clinical and immunologic phenotypes in these patients.

**Table 4:** Distribution of MHC haplotypes among unsolved CVID patients vs. monogenic CVID patients (significant haplotypes with frequency ≥5% are shown).

**MHC-B**	**MHC-E**	**MHC-W**	**MHC-DMA**	**MHC-DMB**	**MHC-TAP1**	**MHC-DPB1**	**MHC-DQB1**	**Monogenic patients (alleles = 80)**	**Unsolved patients (alleles = 86)**	**OR**	***P*-value**
B^*^35		W^*^01:01:01	DMA^*^01:01:01		TAP1^*^01:01:01			6	16	0.40	0.01^*^
B^*^35		W^*^01:01:01	DMA^*^01:01:01	DMB^*^01:03:01:02	TAP1^*^01:01:01			0	7	NI	0.003^**^
B^*^35							DQB1^*^03:01:01	2	9	0.23	0.01^*^
B^*^35						DPB1^*^04:01:01		1	7	0.15	0.01^*^
B^*^35				DMB^*^01:03:01:02				0	7	NI	0.003^**^
	E^*^01:01:01:04	W^*^01:01:01	DMA^*^01:01:01		TAP1^*^01:01:01			2	9	0.23	0.01^*^
	E^*^01:01:01:04					DPB1^*^04:01:01		0	8	NI	0.002^**^
		W^*^01:01:01	DMA^*^01:01:01	DMB^*^01:03:01:02	TAP1^*^01:01:01			0	11	NI	<0.001^***^
		W^*^01:01:01	DMA^*^01:01:01		TAP1^*^01:01:01	DPB1^*^04:01:01		0	9	NI	0.001^**^
		W^*^01:01:01	DMA^*^01:01:01		TAP1^*^01:01:01	DPB1^*^04:01:01	DQB1^*^03:01:01	0	6	NI	0.007^**^
		W^*^01:01:01	DMA^*^01:01:01		TAP1^*^01:01:01		DQB1^*^03:01:01	2	10	0.21	0.007^**^
						DPB1^*^04:01:01	DQB1^*^03:01:01	0	6	NI	0.007^**^

*OR, odds ratio; NI, not calculable*.

* Statistically significant difference, p < 0.05,

** p < 0.01,

*** *p < 0.001*.

**Figure 2:**
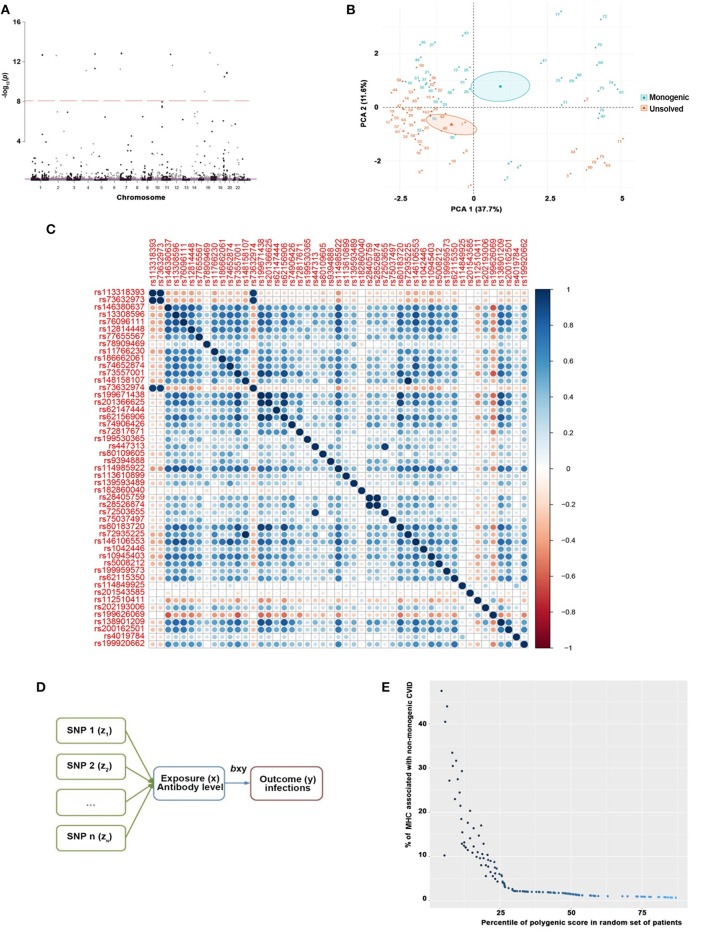
**(A)** Manhattan plot of exome-wide significant results of 535,486 SNPs between monogenic and unsolved CVID patients supporting a polygenic etiology of unsolved CVID where variants with a genotyping rate <98%, ambiguitious SNPs, evidence of deviation from Hardy-Weinberg equilibrium in controls (*p* < 1 × 10^−4^), and minor allele frequency < 1 × 10^−6^ were removed. Only the near-independent SNPs that do not account for linkage disequilibrium and were significantly different between monogenic and unsolved patients were included for the next step. **(B)** Principal component analysis (PCA) and **(C)** correlation matrix of significant variants discriminating monogenic and non-monogenic CVID patients. **(D)** MR model generated for selected variants were inputed to a considering them as direct linked with exposure (antibody deficiency) and outcome (infectious diseases). **(E)** Polygenic score of unsolved CVID disease was increased when the testing dataset had a lower percentage of MHC risk alleles.

Exome-wide significant results between monogenic and unsolved CVID patients and variants with the strongest association compared to healthy individuals were selected for MR analysis. The distribution of significant variants supported a polygenic etiology of unsolved CVID (Figure 2A). Correlation matrix and principal component analysis (PCA) of selected variants showed two distinct sets of SNPs, discriminating monogenic and unsolved CVID patients (Figures 2B,C). Subsequently, the selected variants were incorporated into a MR model considering them as directly linked to antibody deficiency (Figure 2D) and compared with previously suggested variants found in GWAS studies of antibody deficient patients without a genetic evaluation (Table S25). Table 5 and Table S26 summarize the MR estimates from each method of the causal effect of the exposures (variants of the current study and previous GWAS studies) on the susceptibility to infectious diseases as an outcome. The Wald ratio effects reported in the previous GWAS studies, neglecting the exclusion of monogenic diseases, showed only a significant negative correlation of IgG levels on streptococcal infection (*p* = 0.002) and IgA levels on bacterial infection (*p* = 0.04). Although our MR analysis approach also only provide a link between unsolved CVID with bacterial infections, all the estimated coefficients for other infectious diseases were directly associated with these SNPs, whereas several discrepancies were have been observed in MR analyses of previous GWAS studies (Table S26).

**Table 5:** MR estimates from each method of the causal effect of the exposures (variants of current studies and GWAS studies) on the infectious diseases as outcome.

**Outcomes**	**MR method**	**β**	**Standard error**	***P*-value**
Lower respiratory infection	MR Egger	0.002238	0.002275	0.358
	Weighted median	0.0001442	0.0001085	0.1838
	Inverse variance weighted	0.00009826	0.00007786	0.2069
	Weighted mode	0.0001789	0.000178	0.3442
Staphylococcal infection	MR Egger	0.00001533	0.0002204	0.9465
	Weighted median	−0.000003872	0.000007605	0.6106
	Inverse variance weighted	−0.000001176	0.00000711	0.8686
	Weighted mode	−0.000003908	0.00001376	0.7835
Bacterial infection	MR Egger	0.002014	0.0001429	0.02016*
	Weighted median	0.00007075	0.000006549	0.028*
	Inverse variance weighted	0.00008677	0.000004927	0.04821*
	Weighted mode	0.00007561	0.0000103	0.04838*
Other bacterial infections	MR Egger	−0.00005373	0.0001598	0.7465
	Weighted median	0.000001372	0.000007065	0.8461
	Inverse variance weighted	−6.593e-7	0.000005509	0.9047
	Weighted mode	0.000004278	0.00001016	0.6848
Streptococcus infection	MR Egger	0.000005027	0.0002213	0.9825
	Weighted median	−0.000005359	0.000007243	0.4594
	Inverse variance weighted	−0.000002277	0.000007138	0.7498
	Weighted mode	−0.000004368	0.00001126	0.7082
Streptococcal infection, unspecific	MR Egger	0.00009312	0.0001238	0.4764
	Weighted median	0.000002508	0.000005664	0.6579
	Inverse variance weighted	0.000002418	0.000004267	0.571
	Weighted mode	0.000002594	0.000008395	0.7652

**Figure 3:**
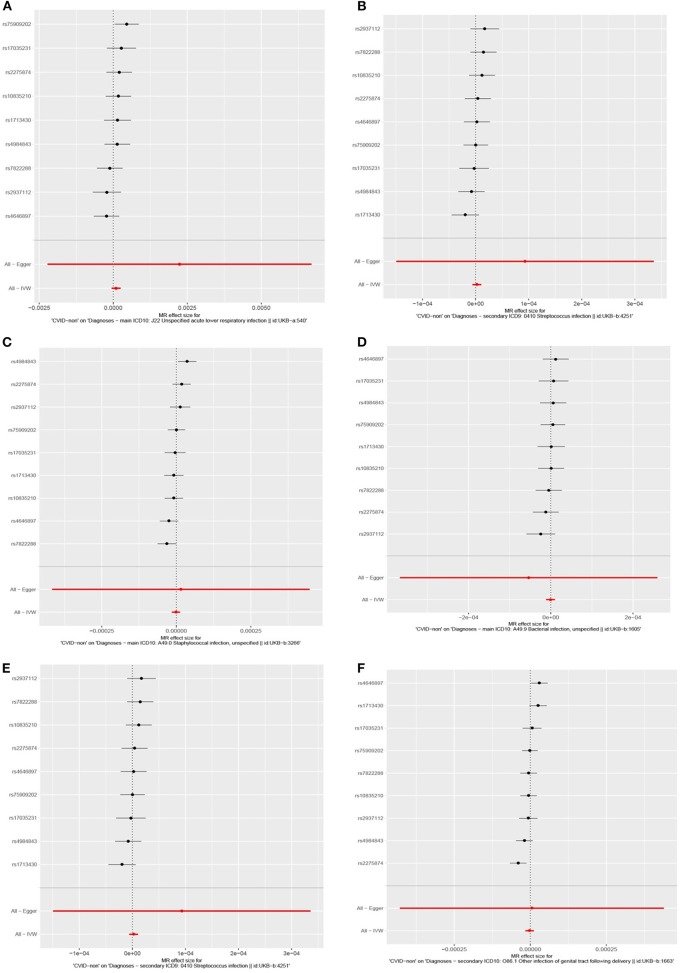
Forest plots depicting causal effect of every single SNP in this cohort by exclusion of monogenic CVID patients suggesting a unified prediction on outcome for all infectious diseases including **(A)** lower respiratory infection, **(B)** staphylococcal infection, **(C)** bacterial infections, **(E)** Streptococcus infection, **(F)** Streptococcal infection, unspecific, except unspecific bacterial infections **(D)**.

To evaluate the consistency of the causal estimate of all SNPs observed, the variability in the estimates obtained for each SNP was calculated and heterogeneity was only significant in association with IgA level and lower respiratory infections in previous GWAS studies (MR Egger, *p* = 0.0089 and inverse variance weighted, *p* = 0.016, Table S27) but none in our suggested model (Table S28). Forest plots showed that the causal effect of every single SNP in previously published GWAS studies had a non-unified prediction on outcome in line with formal MR estimates of heterogeneity as shown in Figures S1, S2. However, the symmetric influence of SNPs selected by the exclusion of monogenic CVID on the outcome was observed in our dataset for all infectious diseases evaluated, except unspecific bacterial infections (Figure 3). Asymmetry and larger spread of β_IV_ in the Funnal plots also suggests a higher heterogeneity and presence of horizontal pleiotropy due to the absence of exclusion of monogenic disease in previous GWAS studies (Figures S3, S4), whereas a homogenous β_IV_ value was observed for lower respiratory infection, bacterial infection, and Streptococcal infection in currently identified SNPs (Figure S5). Of note, the polygenic score of unsolved CVID disease was increased when the testing dataset had a lower percentage of MHC risk factor (Figure 2E).

## Discussion

In this study, we demonstrated the importance of exclusion of monogenic CVID on the evaluation of the effects of MHC alleles and polygenic allele scores in MR analyses in the remaining patients. Since the late 1970s, when the association of MHC locus was initially determined as a principal genetic factor in antibody deficient patients (28, 29), the debate still is ongoing about the direct (the presentation of antigens by MHC) or indirect (association with the adjacent gene within the same region) role of these markers on the pathogenesis of these diseases. However, recently, several monogenic diseases originating from genes outside chromosome 6, the genomic location of MHC, have been discovered to underlie these disorders (2–4, 30–33). For the first time, we used this patient group as a control which may deconfound the effect of other genetic etiologies and may identify the difference in MHC markers between patients with and without monogenic defects. Moreover, using high-resolution MHC typing we also investigated both minor and major histocompatibility complex genes which have not been performed hitherto.

The distribution of major histocompatibility alleles in the current study, compared to our previous investigation on a mixed CVID patient population using low-throughput PCR-based molecular DNA typing, provides reproducible data on MHC class II (with proportional increase in DQB1^*^0201, DQA1^*^0103, and DRB1^*^15 alleles) (11), indicating the advantage of next-generation sequencing by generating data on a larger patients sample with a high resolution of the MHC typing. The findings of the current investigation also conferred that despite some observed significant differences between mixed CVID populations and healthy controls (DRB1^*^4, DRB1^*^11, DRB1^*^07), the design of previous studies has led to false positivity in six alleles and false negativity in 14 alleles associated with MHC class II of unsolved CVID (11). Therefore, subtracting the monogenic patients is an essential factor that should be considered when evaluating whether inheritance of a particular MHC haplotype is associated with CVID development.

In a study designed by Waldrep et al. (12) to test for the possibility of synergy (epistasis) between a mutant transmembrane activator and calcium-modulator and cyclophilin-ligand interactor (TACI) and genes located near the MHC class I locus, they stratified patients based on the variants identified in the *TNFRSF13B* gene. Although the strength of the study was not sufficient and only evaluated the polymorphic region of the *TNFRSF13B* gene (exons 3 and 4), their preliminary data support the hypothesis that the overall pattern of MHC alleles in individuals with a mutated TACI allele is different than in individuals with idiopathic CVID. This notion is consistent with the observation in our study by excluding all monogenic disorders underlying CVID and evaluation of both MHC class I and II alleles.

Most of the previous works on genetic susceptibility factors in CVID patients have only focused on MHC class II due to its known role in antibody class switching and affinity maturation. Using a limited number of CVID patients, we showed previously that MHC haplotypes, including DRB1^*^04-DQB1^*^03:01-DQA1^*^03:01 and DRB1^*^01:01-DQB1^*^03:01-DQA1^*^05:05 confer susceptibility to CVID, while DRB1^*^07-DQA1^*^02:01 constitutes a protective haplotype (11). A restricted diversity of MHC class II, in particular, MHC-DR, has also been reported previously in patients with a familial form of CVID with first degree relatives showing IgA deficiency (DRB1^*^03:01-DQB1^*^02:01 and DRB1^*^04) (34–36).

Both MHC class I and II markers in CVID patients could predict the clinical presentation and immunologic profile including enteropathy and autoimmunity (DQ^*^02:05 and DQ^*^8) (13), chronic inflammation (A^*^29) (37), severe infectious complication (A^*^11 and B^*^44) (38, 39), progressive disease (A^*^24, DQB1^*^03:01 and DQA1^*^05:01) (40, 41), and number of marginal zone-like B cells and switched memory B cells (B^*^8 and B^*^44) (42). However, MHC class I and other minor histocompatibility variants in this region have not been evaluated in most previous CVID studies. In a few reports, the effect of an increased proportion of A^*^24, B^*^14, A^*^02-B^*^40, A^*^02-B^*^044, A^*^03-B^*^07, A^*^01-B^*^08:01, and B^*^44-C^*^16, and a reduced proportion of B^*^62 and C^*^7 on the function of NK cells in CVID patients have been demonstrated (43–46). One previous study has also suggested that the CVID risk is increased in patients where Killer cell immunoglobulin-like receptors (KIR)/MHC class I combinations facilitate NK cell activation (B^*^44-KIR3DS1 and C^*^16-KIR2DL3) (46).

With a full resolution MHC typing, we identified a hitherto not recognized, novel MHC haplotype (W^*^01:01:01-DMA^*^01:01:01-DMB^*^01:03:01:02-TAP1^*^01:01:01), which is associated with unsolved CVID in patients with a late onset of symptoms, present a non-progressive form of the disease with an infections only phenotype and post-germinal center defects. Of note, the proportion of some specific MHC markers was also increased in the monogenic CVID patients which could suggest the deprivation of non-monogenic patients from those MHC alleles or it may be due to linkage of MHC markers with monogenic disorders. However, the latter is less likely since the 40 patients included in this study with the monogenic disease have 26 different genetic problems (2).

We also performed a simulation study on common genetic variants with significant differences between monogenic and unsolved CVID patients as an instrument in MR and compared them to previous GWAS studies. The data supports the notion that variants included in our model satisfy the assumptions of Ig production and bacterial infections as instrumental variables. Since the observed SNPs were consistent for prediction of infectious diseases, the cohort sample bias was not significant. Although the effect of sample size and usage of these unweighted allele scores may influence the predictivity and significance of the genetic model, where exclusion of monogenic forms of CVID unified the effect direction on all evaluated outcomes. Although imprecisely weighted allele scores would not bias our observed estimates, we suggest further cross-validation with other CVID cohorts before integration of weights in the MR data analysis to prevent reduction of power.

Since the number of SNPs used for instruments do not influence the effect size, we have included all significant markers discriminating between monogenic and unsolved CVIDs. It has been previously recommended that variants should be selected on the basis of scientific knowledge rather than statistical testing. However, in a majority of previous MR studies, all variants which can be reasonably assumed to be valid instruments have been considered during analysis to improve the precision of the causal estimate. However, we also performed a pathway analysis for instrumented SNPs representing their function in the immune system, particularly O-glycosylation of proteins and TNFR1-induced proapoptotic signaling, which are important for immunoglobulin production (Table S29).

We have also demonstrated that the use of multiple genetic variants in the context of MR has a significant impact on the strength of predictive MHC markers, with slight reductions in power of polygenic scores in CVID carriers of significant MHC markers. These findings support the notion of two separate mechanisms of MHC and polygenic variants in individuals with unsolved CVID. MHC haplotype is a genetic susceptibility factor for CVID which has been identified as a modifying factor for clinical presentation and immunologic phenotype. However, with current findings, we suggest MHC typing and polygenic evaluation of unsolved CVID patients should be integrated into the flowcharts of genetic screening (2). Moreover, with finding population-specific MHC and SNP predictors we can identify the at-risk asymptomatic individuals within the families of CVID patients and follow them up to improve the prognosis of the disease.

Considering the limitation of the current data of using multiple genetic variants, allele scores in particular, and missing data leading to reduced sample sizes for analysis, future studies in multiple variant setting in the whole genomic level and with higher samples size of monogenic and unsolved patients (from a similar population) could help imputation as it has been shown to be effective against any reduction in power due to missing data. The generalization of discovered MHC and SNP markers in unsolved patients should be performed with caution considering the genetic variations of the cohort population (mainly early-onset, higher rate of consanguinity and lower delay in diagnosis may be due to earlier and severe presentation), however, the methodology presented in the current study would be a commonly recommended approach after performing next-generation sequencing. Although familial cases were slightly higher in a group of patients with identified genetic defects, we cannot conclude at least from our data that multiple cases in a family suggest absolutely the monogenic form of CVID, the fact which is consistent with data from several Western cohorts of patients with accumulation of IgAD and CVID in a family without defining underlying genetic defect but similar MHC markers.

Although the exclusion of monogenic disorders in a newly clinically diagnosed CVID patient is a first mandatory step toward evaluation of other pathogenic mechanisms, detection of the causative genomic element is a challenging task in the MHC region in idiopathic patients due to its complexity and density of genes. Based on current data both MHC alleles and their adjacent genes are involved directly or indirectly in the etiology of some of unsolved CVID patients. Therefore, full sequencing of the MHC region in large populations of CVID patients is recommended. For patients with monogenic diseases, MHC typing may also unravel some markers for variation of the clinical presentation in patients with the same mutation or as a predictive marker for morbidity and mortality. However, to prove this we need a global effort to access a significant amount of patients with unique gene defects or better with a unique mutation within a specific gene. Moreover, the current findings indicate the probability of poly-genic etiology in idiopathic CVID patients. However, before extrapolating the polygenic scores observed in these patients, application of instrumental variable methods with genetic instruments to estimate the causal effect of reduction of immunoglobulin levels (rather than other defects in immune or non-immune system) on the infection diseases should be evaluated further with future observational data.

## Data Availability Statement

All datasets generated for this study are included in the article/Supplementary Material.

## Ethics Statement

The studies involving human participants were reviewed and approved by the Karolinska Institutet and Tehran University of Medical Sciences. Written informed consent to participate in this study was provided by the participants and/or their legal guardian/next of kin.

## Author Contributions

HA designed the project, collected the clinical data, interpreted the analysis, and wrote the paper. CL performed the bioinformatic analysis and analyzed and interpreted the data. AA collected the clinical materials and followed up the patients. LH designed the project, analyzed and interpreted the data, and wrote the paper.

### Conflict of Interest

The authors declare that the research was conducted in the absence of any commercial or financial relationships that could be construed as a potential conflict of interest. The handling editor declared a past co-authorship with the authors HA, LH, and CL.
