# Supplementary material for: Histocompatibility Complex Status and Mendelian Randomization Analysis in Unsolved Antibody Deficiency
Source: Front Immunol. 2020 Jan 24;11:14. doi: 10.3389/fimmu.2020.00014 (PMC6993084; doi:10.3389/fimmu.2020.00014)

Supplementary Data

Histocompatibility Complex Status and Mendelian Randomization Analysis in Unsolved Antibody Deficiency

Abolhassani et al.

Table S1- Distribution of MHC-A alleles associated with monogenic and unsolved CVID patients

A	Monogenic patients (n=40/alleles=80)	Unsolved patients (n=43/ alleles=86)	OR	*P*-value
A*01	5	9	0.59	0.16
A*01:01:01:01	4	9	0.47	0.09
A*01:11N	1	0	NI	0.14
A*02	17	13	1.40	0.15
A*02:01	9	10	0.96	0.46
A*02:01:01:01	5	9	0.59	0.16
A*02:01:01:04	1	0	NI	0.14
A*02:01:01:08	1	1	1.075	0.47
A*02:01:01:17	2	0	NI	0.07
A*02:02:01:01	1	0	NI	0.14
A*02:03:01	1	0	NI	0.14
A*02:05:01:01	4	0	NI	0.017*
A*02:06:01:02	1	0	NI	0.14
A*02:09	0	1	NI	0.16
A*02:11:01	0	1	NI	0.16
A*02:657	0	1	NI	0.16
A*02:662	1	0	NI	0.14
A*03	8	12	0.71	0.21
A*03:01:01:01	6	6	1.07	0.44
A*03:02:01	2	6	0.35	0.07
A*11	8	6	1.43	0.24
A*11:01:01:01	7	4	1.88	0.14
A*11:254	0	1	NI	0.16
A*11:50Q	1	0	NI	0.14
A*11:69N	0	1	NI	0.16
A*23:01:01	13	9	1.55	0.13
A*23:01:01:01	0	4	NI	0.16
A*23:01:01:02	1	0	NI	0.14
A*24	18	10	1.93	0.03*
A*24:02	15	9	1.79	0.06
A*24:02:01:01	12	5	2.58	0.02*
A*24:02:01:02L	3	2	1.61	0.29
A*24:02:01:04	0	2	NI	0.08
A*24:03:01:01	1	0	NI	0.14
A*24:11N	0	1	NI	0.16
A*24:152	1	0	NI	0.14
A*24:61	1	0	NI	0.14
A*25:01:01:01	0	1	NI	0.16
A*26	4	7	0.61	0.20
A*26:01:01:01	3	6	0.53	0.17
A*26:08	1	1	1.07	0.47
A*29:01:01:01	2	2	1.07	0.47
A*30:01:01	4	2	2.15	0.17
A*31:01:02:01	1	3	0.35	0.17
A*32	6	7	0.92	0.43
A*32:01:01:01	5	7	0.76	0.31
A*32:02	1	0	NI	0.14
A*33	1	6	0.17	0.03*
A*33:01:01:01	1	3	0.35	0.17
A*33:03:01	0	3	NI	0.04*
A*66:01:01:01	0	1	NI	0.16
A*68	4	3	1.43	0.31
A*68:01:01:02	4	0	NI	0.017*
A*68:02:01:01	0	3	NI	0.04*
A*69:01:01:01	1	0	NI	0.14

OR: odds ratio, NI: not calculable.
***** *Statistically significant difference, p< 0.05.*

Table S2- Distribution of MHC-B alleles associated with monogenic and unsolved CVID patients

B	Monogenic patients (n=40/alleles=80)	Unsolved patients (n=43/ alleles=86)	OR	*P*-value
B*07:02	0	3	NI	0.04*
B*07:02:01:02	0	2	NI	0.08
B*07:02:03	0	1	NI	0.16
B*07:05:01	1	1	1.07	0.47
B*07:05:01:02	1	0	NI	0.14
B*07:05:01:04	0	1	NI	0.16
B*08:01:01	3	3	1.07	0.46
B*08:01:01:02	1	1	1.07	0.47
B*08:01:01:04	1	2	0.53	0.30
B*08:01:01:05	1	0	NI	0.14
B*08:56:02	1	0	NI	0.14
B*13	5	4	1.34	0.32
B*13:01:01	1	0	NI	0.14
B*13:02:01:03	3	4	0.80	0.38
B*13:02:03	1	0	NI	0.14
B*14	3	3	1.07	0.46
B*14:01:01:01	1	0	NI	0.14
B*14:02	1	3	0.35	0.17
B*14:02:01:02	1	1	1.07	0.47
B*14:02:01:03	0	2	NI	0.08
B*14:02:11	1	0	NI	0.14
B*15:17:01:01	3	1	3.22	047
B*18	4	3	1.43	0.31
B*18:01	2	2	1.07	0.47
B*18:01:01:02	1	1	1.07	0.47
B*18:01:01:03	1	0	NI	0.14
B*18:01:25	0	1	NI	0.16
B*18:02	0	1	NI	0.16
B*18:14	1	0	NI	0.14
B*18:69	1	0	NI	0.14
B*27:05	0	2	NI	0.08
B*27:05:02:03	0	1	NI	0.16
B*27:05:18	0	1	NI	0.16
B*35	11	20	0.59	0.05*
B*35:01	4	7	0.61	0.20
B*35:01:01:01	0	1	NI	0.16
B*35:01:01:02	3	4	0.80	0.38
B*35:01:01:03	0	1	NI	0.16
B*35:01:01:07	1	1	1.07	0.47
B*35:02:01:01	4	1	4.3	0.07
B*35:03	1	6	0.17	0.03*
B*35:03:01:03	0	1	NI	0.16
B*35:03:01:04	0	2	NI	0.08
B*35:03:01:05	1	0	NI	0.14
B*35:03:19	0	3	NI	0.04*
B*35:08:01:01	0	3	NI	0.04*
B*35:279	1	0	NI	0.14
B*35:298	0	2	NI	0.08
B*35:41	1	1	1.07	0.47
B*38	6	2	3.22	0.05*
B*38:09	1	1	1.07	0.47
B*38:24	1	1	1.07	0.47
B*38:60	4	0	NI	0.017*
B*39	0	4	NI	0.02*
B*39:01:01:06	0	1	NI	0.16
B*39:06:02:01	0	1	NI	0.16
B*39:10:01	0	1	NI	0.16
B*39:38Q	0	1	NI	0.16
B*40:06:01:02	3	2	1.61	0.29
B*41:01:01	2	5	0.43	0.14
B*44	3	1	3.22	0.13
B*44:02:01:01	1	0	NI	0.14
B*44:03:01:04	0	1	NI	0.16
B*44:03:01:08	1	0	NI	0.14
B*44:06	1	0	NI	0.14
B*45	2	0	NI	0.07
B*45:01:01	1	0	NI	0.14
B*45:04	1	0	NI	0.14
B*49	2	4	0.53	0.22
B*49:01:01	2	1	2.15	0.25
B*49:32	0	1	NI	0.16
B*49:38	0	2	NI	0.08

B*50:01:01	4	4	1.07	0.45
B*50:01:01:01	0	4	NI	0.02*
B*50:01:01:02	4	0	NI	0.017*
B*51	9	10	0.96	0.46
B*51:01	5	6	0.89	0.42
B*51:01:01	2	2	1.07	0.47
B*51:01:01:14	1	1	1.07	0.47
B*51:01:01:15	0	1	NI	0.16
B*51:01:01:20	1	0	NI	0.14
B*51:01:05	3	4	0.80	0.38
B*51:05	2	1	2.15	0.25
B*51:07:01	1	1	1.07	0.47
B*51:187	0	2	NI	0.08
B*51:56:03	1	0	NI	0.14
B*52	5	6	0.89	0.42
B*52:01:01	4	5	0.86	0.40
B*52:01:01:01	0	1	NI	0.16
B*52:01:01:02	2	4	0.53	0.22
B*52:01:01:04	2	0	NI	0.07
B*52:43	1	1	1.07	0.47
B*53:01:01	1	1	1.07	0.47
B*55	4	3	1.43	0.31
B*55:01:01	4	1	4.3	0.07
B*55:02:01:01	0	1	NI	0.16
B*55:85	0	1	NI	0.16
B*56:01:01:04	1	0	NI	0.14
B*57	2	1	2.15	0.25
B*57:29	1	1	1.07	0.47
B*57:86	1	0	NI	0.14
B*58	3	0	NI	0.03*
B*58:01:01:02	1	0	NI	0.14
B*58:01:01:04	1	0	NI	0.14
B*58:01:19	1	0	NI	0.14
B*78:02:02	1	0	NI	0.14

OR: odds ratio, NI: not calculable.
***** *Statistically significant difference, $p < 0.05$.*

Table S3- Distribution of MHC-C alleles associated with monogenic and unsolved CVID patients

C	Monogenic patients (n=40/alleles=80)	Unsolved patients (n=43/ alleles=86)	OR	*P*-value
C*01:03	1	1	1.07	0.47
C*01:106	1	1	1.07	0.47
C*01:30	1	0	NI	0.14
C*01:67	1	0	NI	0.14
C*02:02:02:01	0	2	NI	0.08
C*03	3	1	3.22	0.13
C*03:02:02:03	1	0	NI	0.14
C*03:03:33	0	1	NI	0.16
C*03:04:01:02	2	0	NI	0.07
C*04	17	23	0.79	0.20
C*04:01:01	15	19	0.84	0.43
C*04:01:01:02	2	3	0.71	0.35
C*04:01:01:03	0	3	NI	0.04*
C*04:01:01:05	1	1	1.07	0.47
C*04:01:01:06	11	12	0.98	0.48
C*04:01:01:12	1	0	NI	0.14
C*04:01:73	1	0	NI	0.14
C*04:166	0	1	NI	0.16
C*04:239	1	0	NI	0.14
C*04:243	0	3	NI	0.04*
C*05:01:01:02	1	1	1.07	0.47
C*06	9	7	1.38	0.24
C*06:02:01	4	2	2.15	0.17
C*06:02:01:02	1	0	NI	0.14
C*06:02:01:08	3	2	1.61	0.29
C*06:155:01:01	2	3	0.71	0.35
C*06:160	1	0	NI	0.14
C*06:188	1	0	NI	0.14
C*06:24	1	2	0.53	0.30
C*07	17	16	1.14	0.33
C*07:01	7	9	0.83	0.35
C*07:01:01	5	8	0.67	0.23
C*07:01:01:02	1	3	0.35	0.17
C*07:01:01:06	1	2	0.53	0.30
C*07:01:01:08	0	2	NI	0.08
C*07:01:01:09	0	1	NI	0.16
C*07:01:01:16	3	0	NI	0.03*
C*07:01:02	2	1	2.15	0.25
C*07:02	4	6	0.71	0.29
C*07:02:01	4	5	0.86	0.40
C*07:02:01:04	1	0	NI	0.14
C*07:02:01:05	1	1	1.07	0.47
C*07:02:01:09	2	3	0.71	0.35
C*07:02:01:12	0	1	NI	0.16
C*07:02:05	0	1	NI	0.16
C*07:04:01:02	2	0	NI	0.07
C*07:18	2	1	2.15	0.25
C*07:547	1	0	NI	0.14
C*07:549	1	0	NI	0.14
C*08	3	2	1.61	0.29
C*08:02:01:02	3	1	3.22	0.13
C*08:12	0	1	NI	0.16
C*12	13	13	1.07	0.42
C*12:02	3	4	0.80	0.38
C*12:02:02	3	3	1.07	0.46
C*12:02:02:01	2	2	1.07	0.47
C*12:02:02:02	1	1	1.07	0.47
C*12:02:12	0	1	NI	0.16
C*12:03:01	8	5	1.72	0.15
C*12:03:01:06	0	1	NI	0.16
C*12:03:01:07	1	0	NI	0.14
C*12:03:01:08	4	1	4.3	0.07
C*12:03:01:09	3	3	1.07	0.46
C*12:12	0	1	NI	0.16
C*12:162	0	1	NI	0.16
C*12:177	1	0	NI	0.14
C*12:178	1	0	NI	0.14
C*12:57:02	0	1	NI	0.16
C*12:73	1	0	NI	0.14
C*12:99:01	0	1	NI	0.16
C*14	2	5	0.43	0.14
C*14:02:01	2	3	0.71	0.35

C*14:02:01:01	2	2	1.07	0.47
C*14:02:01:03	0	1	NI	0.16
C*14:03	0	1	NI	0.16
C*14:69	0	1	NI	0.16
C*15	5	8	0.67	0.23
C*15:02:01:01	4	6	0.71	0.29
C*15:05:02	0	1	NI	0.16
C*15:103	0	1	NI	0.16
C*15:104	1	0	NI	0.14
C*16	3	1	3.22	0.13
C*16:02:01	2	0	NI	0.07
C*16:04:01:01	1	1	1.07	0.47
C*17	2	5	0.43	0.14
C*17:01:01:05	2	4	0.53	0.22
C*17:03:01:01	0	1	NI	0.16

OR: odds ratio, NI: not calculable.

***** *Statistically significant difference, $p < 0.05$.*

Table S4- Distribution of MHC-E alleles associated with monogenic and unsolved CVID patients

**E**	**Monogenic patients (n=40/alleles=80)**	**Unsolved patients (n=43/ alleles=86)**	**OR**	***P*-value**
E*01:01	32	40	0.86	0.19
E*01:01:01:01	9	3	3.22	0.02*
E*01:01:01:03	1	1	1.07	0.47
E*01:01:01:04	9	19	0.50	0.03*
E*01:01:01:05	8	9	0.95	0.46
E*01:01:01:06	0	2	NI	0.08
E*01:01:01:07	2	4	0.53	
E*01:01:01:08	3	2	1.61	0.29
E*01:03	46	40	1.23	0.07
E*01:03:01:01	1	0	NI	0.14
E*01:03:01:02	9	13	0.74	0.23
E*01:03:01:03	8	3	2.86	0.04*
E*01:03:01:04	26	22	1.27	0.16
E*01:03:02:02	0	2	NI	0.08
E*01:03:04	2	0	NI	0.07
E*01:08N	0	4	NI	0.02*
E*01:09	2	2	1.07	0.47

OR: odds ratio, NI: not calculable.
***** *Statistically significant difference, p< 0.05.*

Table S5- Distribution of MHC-F alleles associated with monogenic and unsolved CVID patients

**F**	**Monogenic patients (n=40/alleles=80)**	**Unsolved patients (n=43/ alleles=86)**	**OR**	***P*-value**
F*01:01	68	68	1.07	0.16
F*01:01:01	54	49	1.18	0.08
F*01:01:01:01	13	7	1.99	0.05*
F*01:01:01:02	0	2	NI	0.08
F*01:01:01:05	3	3	1.07	0.46
F*01:01:01:08	22	16	1.47	0.08
F*01:01:01:09	12	20	0.64	0.08
F*01:01:01:12	4	1	4.3	0.07
F*01:01:02	14	19	0.79	0.22
F*01:01:02:04	3	9	0.35	0.04*
F*01:01:02:06	11	10	1.18	0.35
F*01:03:01:01	10	17	0.63	0.10
F*01:04	2	1	2.15	0.25

OR: odds ratio, NI: not calculable.

***** *Statistically significant difference, p< 0.05.*

Table S6- Distribution of MHC-G alleles associated with monogenic and unsolved CVID patients

**G**	**Monogenic patients (n=40/alleles=80)**	**Unsolved patients (n=43/ alleles=86)**	**OR**	***P*-value**
G*01:01	46	58	0.85	0.09
G*01:01:01	24	30	0.86	0.25
G*01:01:01:01	1	1	1.07	0.47
G*01:01:01:02	14	15	1.00	0.49
G*01:01:01:04	1	1	1.07	0.47
G*01:01:01:05	7	12	0.62	0.14
G*01:01:01:06	0	1	NI	0.16
G*01:01:01:07	1	0	NI	0.14
G*01:01:02	10	17	0.63	0.10
G*01:01:02:01	10	16	0.67	0.13
G*01:01:02:02	0	1	NI	0.16
G*01:01:03:03	8	6	1.43	0.24
G*01:01:12	4	5	0.86	0.40
G*01:03:01:02	7	3	2.50	0.07
G*01:04	17	15	1.21	0.26
G*01:04:01	14	11	1.36	0.19
G*01:04:03	2	0	NI	0.07
G*01:04:04	1	4	0.26	0.10
G*01:05N	4	2	2.15	0.17
G*01:06	6	7	0.92	0.43
G*01:08:01	0	2	NI	0.08

OR: odds ratio, NI: not calculable.

***** *Statistically significant difference, p< 0.05.*

Table S7- Distribution of MHC-H alleles associated with monogenic and unsolved CVID patients

H	Monogenic patients (n=40/alleles=80)	Unsolved patients (n=43/ alleles=86)	OR	*P*-value
H*01:01	25	28	0.95	0.42
H*01:01:01:01	18	14	1.38	0.15
H*01:02	7	14	0.53	0.07
H*02	55	58	1.01	0.42
H*02:01:01:01	26	21	1.33	0.12
H*02:03	29	37	0.84	0.18

OR: odds ratio, NI: not calculable.
***** *Statistically significant difference, $p< 0.05$.*

Table S8- Distribution of MHC-W alleles associated with monogenic and unsolved CVID patients

**W**	**Monogenic patients (n=40/alleles=80)**	**Unsolved patients (n=43/ alleles=86)**	**OR**	***P*-value**
W*01:01:01	43	58	0.79	0.03*
W*01:01:01:01	10	13	0.82	0.31
W*01:01:01:02	6	10	0.64	0.18
W*01:01:01:04	9	14	0.69	0.17
W*01:01:01:05	11	12	0.98	0.48
W*01:01:01:06	7	9	0.83	0.35
W*02:01	3	2	1.61	0.29
W*03:01:01	24	22	1.17	0.26
W*03:01:01:01	15	13	1.24	0.26
W*03:01:01:02	9	9	1.07	0.43
W*04:01	9	7	1.38	0.24

OR: odds ratio, NI: not calculable.
***** *Statistically significant difference, p< 0.05.*

Table S9- Distribution of MHC-DMA alleles associated with monogenic and unsolved CVID patients

**DMA**	**Monogenic patients (n=40/alleles=80)**	**Unsolved patients (n=43/ alleles=86)**	**OR**	***P*-value**
DMA*01:01:01	50	66	0.81	0.02*
DMA*01:01:01:01	1	3	0.35	0.17
DMA*01:01:01:02	17	23	0.79	0.20
DMA*01:01:01:03	21	19	1.18	0.26
DMA*01:01:01:04	11	21	0.56	0.04*
DMA*01:02	29	20	1.55	0.03*

OR: odds ratio, NI: not calculable.

***** *Statistically significant difference, p< 0.05.*

Table S10- Distribution of MHC-DMB alleles associated with monogenic and unsolved CVID patients

DMB	Monogenic patients (n=40/alleles=80)	Unsolved patients (n=43/ alleles=86)	OR	*P*-value
DMB*01:01:01	70	69	1.09	0.10
DMB*01:01:01:01	25	29	0.92	0.36
DMB*01:01:01:02	21	24	0.94	0.40
DMB*01:01:01:03	16	10	1.72	0.06
DMB*01:01:01:04	8	6	1.43	0.24
DMB*01:03:01	10	17	0.63	0.10
DMB*01:03:01:01	5	0	NI	0.009**
DMB*01:03:01:02	5	17	0.31	0.005**

OR: odds ratio, NI: not calculable.

***** *Statistically significant difference, p< 0.05.*

Table S11- Distribution of MHC-DOA alleles associated with monogenic and unsolved CVID patients

**DOA**	**Monogenic patients (n=40/alleles=80)**	**Unsolved patients (n=43/ alleles=86)**	**OR**	***P*-value**
DOA*01:01:01	14	15	1.00	0.49
DOA*01:01:02	34	47	0.77	0.08
DOA*01:01:02:01	5	10	0.53	0.11
DOA*01:01:02:02	26	36	0.77	0.10
DOA*01:01:02:03	3	1	3.22	0.13
DOA*01:01:04	18	18	1.07	0.26
DOA*01:01:04:01	17	17	1.07	0.40
DOA*01:01:04:02	1	1	1.07	0.47
DOA*01:01:05	14	6	2.50	0.01*

OR: odds ratio, NI: not calculable.

***** *Statistically significant difference, p< 0.05.*

Table S12- Distribution of MHC-DOB alleles associated with monogenic and unsolved CVID patients

**DOB**	**Monogenic patients (n=40/alleles=80)**	**Unsolved patients (n=43/ alleles=86)**	**OR**	***P*-value**
DOB*01	63	74	0.91	0.10
DOB*01:01:01	40	52	0.82	0.08
DOB*01:01:01:01	24	33	0.78	0.12
DOB*01:01:01:02	2	0	NI	0.07
DOB*01:01:01:03	1	2	0.53	0.30
DOB*01:01:01:04	13	17	0.82	0.27
DOB*01:01:03:02	23	22	1.12	0.32
DOB*01:02:02	4	0	NI	0.01*
DOB*01:04:01:02	3	2	1.61	0.29
DOB*01:05	10	10	1.07	0.43

OR: odds ratio, NI: not calculable.

***** *Statistically significant difference, p< 0.05.*

Table S13- Distribution of MHC-DPA alleles associated with monogenic and unsolved CVID patients

**DPA**	**Monogenic patients (n=40/alleles=80)**	**Unsolved patients (n=43/ alleles=86)**	**OR**	***P*-value**
DPA1*01	58	67	0.93	0.20
DPA1*01:03:01	41	51	0.86	0.14
DPA1*01:03:01:01	12	13	0.99	0.49
DPA1*01:03:01:02	15	13	1.24	0.26
DPA1*01:03:01:03	2	6	0.35	0.08
DPA1*01:03:01:04	2	13	0.16	0.002**
DPA1*01:03:01:05	10	6	1.79	0.11
DPA1*01:14	17	16	1.14	0.33
DPA1*02	22	19	1.24	0.20
DPA1*02:01	17	15	1.21	0.26
DPA1*02:01:01:01	16	15	1.14	0.33
DPA1*02:01:02	1	0	NI	0.14
DPA1*02:02:02	0	2	NI	0.08
DPA1*02:07:01:01	5	2	2.68	0.10

OR: odds ratio, NI: not calculable.

***** *Statistically significant difference, p< 0.05.*

Table S14- Distribution of MHC-TAP1 alleles associated with monogenic and unsolved CVID patients

**TAP1**	**Monogenic patients (n=40/alleles=80)**	**Unsolved patients (n=43/ alleles=86)**	**OR**	***P*-value**
TAP1*01:01:01	75	86	0.94	0.009**
TAP1*01:01:01:01	26	47	0.60	0.002**
TAP1*01:01:01:02	19	22	0.92	0.39
TAP1*01:01:01:03	10	7	1.53	0.17
TAP1*01:01:01:05	20	10	2.15	0.01*
TAP1*06:01	5	0	NI	0.009**

OR: odds ratio, NI: not calculable.

***** *Statistically significant difference, $p < 0.05$.*

Table S15- Distribution of MHC-TAP2 alleles associated with monogenic and unsolved CVID patients

TAP2	Monogenic patients (n=40/alleles=80)	Unsolved patients (n=43/ alleles=86)	OR	*P*-value
TAP2*01	53	55	1.03	0.37
TAP2*01:01	51	46	1.19	0.09
TAP2*01:01:02	19	16	1.27	0.20
TAP2*01:01:03	32	30	1.14	0.24
TAP2*01:01:03:01	9	11	0.87	0.38
TAP2*01:01:03:02	16	13	1.32	0.20
TAP2*01:01:03:03	7	6	1.25	0.33
TAP2*01:04	2	9	0.23	0.01*
TAP2*02:01:02	27	31	0.93	0.37
TAP2*02:01:02:01	11	6	1.97	0.07
TAP2*02:01:02:02	0	5	NI	0.01*
TAP2*02:01:02:03	16	20	0.86	0.30

OR: odds ratio, NI: not calculable.

***** *Statistically significant difference, p< 0.05.*

Table S16- Distribution of MHC-DPB1 alleles associated with monogenic and unsolved CVID patients

DPB1	Monogenic patients (n=40/alleles=80)	Unsolved patients (n=43/ alleles=86)	OR	*P*-value
DPB1*01:01:02	4	3	1.43	0.31
DPB1*01:01:02:01	2	0	NI	0.07
DPB1*01:01:02:02	2	3	0.71	0.35
DPB1*02:01:02	8	12	0.71	0.21
DPB1*02:01:02:10	1	2	0.53	0.30
DPB1*02:01:02:13	5	9	0.59	0.16
DPB1*02:01:02:16	2	1	2.15	0.25
DPB1*03:01:01	2	2	1.07	0.47
DPB1*03:01:01:01	2	0	NI	0.07
DPB1*03:01:01:04	0	2	NI	0.08
DPB1*04:01:01	6	16	0.40	0.01*
DPB1*04:01:01:01	1	5	0.21	0.03*
DPB1*04:01:01:03	2	3	0.71	0.35
DPB1*04:01:01:08	3	8	0.40	0.07
DPB1*04:02:01	3	2	1.61	0.29
DPB1*04:02:01:02	1	1	1.07	0.47
DPB1*04:02:01:03	1	1	1.07	0.47
DPB1*04:02:01:05	1	0	NI	0.14
DPB1*05:01:01	1	4	0.26	0.01
DPB1*05:01:01:01	1	2	0.53	0.30
DPB1*05:01:01:02	0	1	NI	0.16
DPB1*05:01:01:03	0	1	NI	0.16
DPB1*09:01:01	2	2	1.07	0.47
DPB1*10:01:01:02	1	0	NI	0.14
DPB1*104:01:01	2	2	1.07	0.47
DPB1*104:01:01:02	2	0	NI	0.07
DPB1*104:01:01:03	0	2	NI	0.08
DPB1*105:01:01	1	2	0.53	0.30
DPB1*105:01:01:01	1	0	NI	0.14
DPB1*105:01:01:03	0	2	NI	0.08
DPB1*13:01:01	6	0	NI	0.004**
DPB1*14:01:01:01	3	1	3.22	0.13
DPB1*15:01:01	3	0	NI	0.03*
DPB1*17:01:01	0	5	NI	0.01**
DPB1*17:01:01:01	0	3	NI	0.04*
DPB1*17:01:01:02	0	2	NI	0.08
DPB1*19:01	0	1	NI	0.16
DPB1*23:01:01	1	1	1.07	0.47
DPB1*260:01	1	0	NI	0.14
DPB1*28:01	9	8	1.20	0.76
DPB1*31:01	6	7	0.92	0.43
DPB1*414:01	0	2	NI	0.08
DPB1*416:01:01:01	1	0	NI	0.14
DPB1*45:01	0	1	NI	0.16
DPB1*463:01:01	13	10	1.39	0.19
DPB1*463:01:01:01	1	0	NI	0.14
DPB1*463:01:01:02	5	5	1.07	0.45
DPB1*463:01:01:03	7	5	1.50	0.23
DPB1*49:01:01:02	1	0	NI	0.14
DPB1*648:01:01:02	6	5	1.29	0.33

OR: odds ratio, NI: not calculable.

***** *Statistically significant difference, p< 0.05.*

Table S17- Distribution of MHC-DQA1 alleles associated with monogenic and unsolved CVID patients

**DQA1**	**Monogenic patients (n=40/alleles=80)**	**Unsolved patients (n=43/ alleles=86)**	**OR**	***P*-value**
DQA1*01:02:01	4	1	4.3	0.17
DQA1*01:02:01:02	3	0	NI	0.03*
DQA1*01:02:01:03	1	1	1.07	0.47
DQA1*01:03:01:01	15	4	4.03	0.002**
DQA1*01:04:01	1	12	0.08	<0.001***
DQA1*01:04:01:01	1	8	0.13	0.01*
DQA1*01:04:01:02	0	4	NI	0.005**
DQA1*01:05	2	8	0.26	0.03*
DQA1*01:05:01	2	7	0.30	0.05*
DQA1*01:05:02	0	1	NI	0.16
DQA1*02:01:01:01	3	4	0.80	0.38
DQA1*03	15	13	1.24	0.26
DQA1*03:01:01	5	6	0.89	0.42
DQA1*03:02	1	1	1.07	0.47
DQA1*03:03:01	9	6	1.61	0.16
DQA1*03:03:01:01	4	4	1.07	0.42
DQA1*03:03:01:02	4	0	NI	0.01*
DQA1*03:03:01:03	1	2	0.53	0.30
DQA1*04	12	19	0.67	0.12
DQA1*04:01	3	3	1.07	0.46
DQA1*04:01:01	1	0	NI	0.14
DQA1*04:01:02:01	0	2	NI	0.08
DQA1*04:01:02:02	2	1	2.15	0.25
DQA1*04:02	9	16	0.60	0.09
DQA1*05	28	25	1.20	0.20
DQA1*05:01:01	10	8	1.34	0.25
DQA1*05:01:01:01	0	2	NI	0.08
DQA1*05:01:01:02	10	6	1.79	0.11
DQA1*05:05:01	17	16	1.14	0.33
DQA1*05:05:01:01	1	1	1.07	0.47
DQA1*05:05:01:02	1	2	0.53	0.30
DQA1*05:05:01:03	1	2	0.53	0.30
DQA1*05:05:01:06	1	1	1.07	0.47
DQA1*05:05:01:07	13	9	1.55	0.17
DQA1*05:05:01:08	0	1	NI	0.16
DQA1*05:09	1	1	1.07	0.47

OR: odds ratio, NI: not calculable.

*** Statistically significant difference, $p < 0.05$.*

Table S18- Distribution of MHC-DQB1 alleles associated with monogenic and unsolved CVID patients

**DQB1**	**Monogenic patients (n=40/alleles=80)**	**Unsolved patients (n=43/ alleles=86)**	**OR**	***P*-value**
DQB1*02	42	38	1.18	0.14
DQB1*02:01:01	7	7	1.07	0.44
DQB1*02:02:01:01	1	2	0.53	0.30
DQB1*02:53Q	5	4	1.34	0.32
DQB1*02:62	8	13	0.66	0.16
DQB1*02:80	5	3	1.79	0.20
DQB1*02:82	0	1	NI	0.16
DQB1*02:83	5	2	2.68	0.10
DQB1*02:84	11	6	1.97	0.07
DQB1*03:01	15	28	0.57	0.02*
DQB1*03:01:01	4	17	0.25	0.002**
DQB1*03:01:01:01	0	4	NI	0.005**
DQB1*03:01:01:02	1	2	0.53	0.30
DQB1*03:01:01:03	1	4	0.26	0.10
DQB1*03:01:01:04	2	4	0.53	0.22
DQB1*03:01:01:05	0	3	NI	0.04*
DQB1*03:02:01	6	7	0.92	0.43
DQB1*03:02:01:01	3	5	0.64	0.26
DQB1*03:02:01:02	3	2	1.61	0.29
DQB1*03:03:02	2	0	NI	0.07
DQB1*03:03:02:01	1	0	NI	0.14
DQB1*03:03:02:04	1	0	NI	0.14
DQB1*03:05:01	2	4	0.53	0.22
DQB1*03:19:01	1	0	NI	0.14
DQB1*04:02:01:01	0	1	NI	0.16
DQB1*05	8	15	0.57	0.08
DQB1*05:01:01	3	6	0.53	0.17
DQB1*05:01:01:01	2	3	0.71	0.35
DQB1*05:01:01:02	1	2	0.53	0.30
DQB1*05:01:01:03	0	1	NI	0.16
DQB1*05:02:01	4	2	2.15	0.17
DQB1*05:03:01:01	1	7	0.15	0.01*
DQB1*06	15	4	4.03	0.004**
DQB1*06:01:01	6	0	NI	0.004**
DQB1*06:02:01:01	1	2	0.53	0.30
DQB1*06:03:01	5	1	5.37	0.03*
DQB1*06:04:01	3	1	3.22	0.13

OR: odds ratio, NI: not calculable.

***** *Statistically significant difference, p< 0.05.*

Table S19- Distribution of MHC-DRB1 alleles associated with monogenic and unsolved CVID patients

DRB1	Monogenic patients (n=40/alleles=80)	Unsolved patients (n=43/ alleles=86)	OR	*P*-value
DRB1*01	2	3	0.71	0.35
DRB1*01:01:01	1	2	0.53	0.30
DRB1*01:02:01	1	1	1.07	0.47
DRB1*03:01:01:01	1	0	NI	0.14
DRB1*04:01:01:01	0	3	NI	0.04*
DRB1*07:01:01	6	8	0.80	0.33
DRB1*07:01:01:01	0	4	NI	0.02*
DRB1*07:01:01:02	6	4	1.61	0.22
DRB1*09:21	9	8	1.20	0.33
DRB1*10:01:01:01	0	1	NI	0.16
DRB1*11	16	9	1.91	0.04*
DRB1*11:01	12	4	3.22	0.01*
DRB1*11:01:01:01	4	3	1.43	0.31
DRB1*11:01:02	8	1	8.6	0.006**
DRB1*11:04:01	4	5	0.86	0.40
DRB1*13	2	2	1.07	0.47
DRB1*13:01:01:01	2	1	2.15	0.25
DRB1*13:02:01	0	1	NI	0.16
DRB1*14	0	6	NI	0.008**
DRB1*14:01:01	0	1	NI	0.16
DRB1*14:54:01	0	5	NI	0.01**
DRB1*15	42	43	1.05	0.37
DRB1*15:01:01	28	35	0.86	0.22
DRB1*15:01:01:01	8	13	0.66	0.16
DRB1*15:01:01:02	8	5	1.72	0.15
DRB1*15:01:01:03	4	11	0.39	0.04*
DRB1*15:01:01:04	8	6	1.43	0.24
DRB1*15:02:01:02	2	2	1.07	0.47
DRB1*15:03:01	12	6	2.15	0.04*
DRB1*15:03:01:01	7	2	3.76	0.03*
DRB1*15:03:01:02	5	4	1.34	0.32
DRB1*16:02:01:02	2	3	0.71	0.35

OR: odds ratio, NI: not calculable.
***** *Statistically significant difference, p< 0.05.*

Table S20- Distribution of MHC-DRB3 alleles associated with monogenic and unsolved CVID patients

DRB3	Monogenic patients (n=40/alleles=80)	Unsolved patients (n=43/ alleles=86)	OR	*P*-value
DRB3*01:01:02:01	27	35	0.82	0.17
DRB3*02:02:01	53	51	1.11	0.17
DRB3*02:02:01:01	38	35	1.16	0.15
DRB3*02:02:01:02	15	16	1.00	0.49

OR: odds ratio, NI: not calculable.

***** *Statistically significant difference, p< 0.05.*

Table S21- Distribution of MHC-DRB4 alleles associated with monogenic and unsolved CVID patients

DRB4	Monogenic patients (n=40/alleles=80)	Unsolved patients (n=43/ alleles=86)	OR	*P*-value
DRB4*01:01:01:01	17	14	1.30	0.20
DRB4*01:03:01	63	72	0.94	0.20
DRB4*01:03:01:01	33	41	0.86	0.20
DRB4*01:03:01:03	30	31	1.04	0.42

OR: odds ratio, NI: not calculable.

***** *Statistically significant difference, p< 0.05.*

Table S22- Distribution of MHC-MICA alleles associated with monogenic and unsolved CVID patients

MICA	Monogenic patients (n=40/alleles=80)	Unsolved patients (n=43/ alleles=86)	OR	*P*-value
MICA*001	16	20	0.86	0.30
MICA*004	14	19	0.79	0.22
MICA*008	50	48	1.11	0.19
MICA*008:01:01	22	20	1.18	0.26
MICA*008:01:02	17	23	0.79	0.20
MICA*008:04	11	5	2.36	0.04*

OR: odds ratio, NI: not calculable.

***** *Statistically significant difference, p< 0.05.*

Table S23- Distribution of MHC-MICB alleles associated with monogenic and unsolved CVID patients

**MICB**	**Monogenic patients (n=40/alleles=80)**	**Unsolved patients (n=43/ alleles=86)**	**OR**	***P*-value**
MICB*002:01	35	17	2.21	<0.001***
MICB*002:01:01	3	4	0.80	0.38
MICB*002:01:02	32	13	2.64	<0.001***
MICB*003	11	13	0.90	0.06
MICB*004:01	12	16	0.80	0.26
MICB*004:01:01	3	7	0.46	0.11
MICB*004:01:02	9	9	1.07	0.43
MICB*005:02	21	30	0.75	0.11
MICB*005:02:01	3	7	0.46	0.11
MICB*005:02:02	2	10	0.21	0.01*
MICB*005:02:03	16	13	1.32	0.20
MICB*005:02:04	0	1	NI	0.16
MICB*008	4	6	0.71	0.29

OR: odds ratio, NI: not calculable.

***** *Statistically significant difference, p< 0.05.*

Table S24- Regression model analysis using the signification MHC allele for prediction of unsolved CVID patients

MICB	Estimate	Standard. Error	T value	*P*-value
Intercept	1.61492	0.08309	19.436	< 2e-16 ***
B*35	-0.26855	0.10087	-2.662	0.00947 **
DMA*01:02	-0.53703	0.21918	-2.450	0.01658 *
TAP1*06:01	0.39020	0.13044	2.991	0.00374 **
MICB*002:01	-0.70178	0.14851	-4.726	1.03e-05 ***
DQA1*01:04:01	0.30561	0.15984	1.912	0.04965*
DQB1*03:01:01	0.37085	0.18295	2.027	0.04617 *

*Signif. codes: 0 '***' 0.001 '**' 0.01 '*' 0.05 '.' 0.1 ' ' 1*
Residual standard error: 0.4145 on 76 degrees of freedom
Multiple R-squared: 0.3768, Adjusted R-squared: 0.3194
F-statistic: 6.565 on 7 and 76 DF, p-value: 4.556e-06

Table S25- Previously suggested SNP associated with antibody production from GWAS studies.

Phenotype_simple	Initial_sample_description	Replication_sample_description	PubmedID	SNP	gene	P-value
**IgA levels**	430 European ancestry cases, 1,090 European ancestry controls	342 European ancestry cases, 886 European ancestry controls	20694011	rs10492294	*PVT1*	0.000004
	430 European ancestry cases, 1,090 European ancestry controls	342 European ancestry cases, 886 European ancestry controls	20694011	rs11038871	*DGKZ*	0.000002
	430 European ancestry cases, 1,090 European ancestry controls	342 European ancestry cases, 886 European ancestry controls	20694011	rs11662763	*L3MBTL4*	0.000005
	430 European ancestry cases, 1,090 European ancestry controls	342 European ancestry cases, 886 European ancestry controls	20694011	rs12669076	*SHFM1*	0.000002
	430 European ancestry cases, 1,090 European ancestry controls	342 European ancestry cases, 886 European ancestry controls	20694011	rs2187668	*MHC-DRB1*	2e-33
	430 European ancestry cases, 1,090 European ancestry controls	342 European ancestry cases, 886 European ancestry controls	20694011	rs2234978	*ACTA2*	0.000006
	430 European ancestry cases, 1,090 European ancestry controls	342 European ancestry cases, 886 European ancestry controls	20694011	rs6498142	*CLEC16A*	2e-7
	430 European ancestry cases, 1,090 European ancestry controls	342 European ancestry cases, 886 European ancestry controls	20694011	rs669408	*SIPA1L2*	0.000001
	430 European ancestry cases, 1,090 European ancestry controls	342 European ancestry cases, 886 European ancestry controls	20694011	rs7029145	*IFNK*	0.000009
	9,617 European ancestry individuals	2,785 European ancestry individuals	24676358	rs7853287	*CD30L*	3e-10
	430 European ancestry cases, 1,090 European ancestry controls	342 European ancestry cases, 886 European ancestry controls	20694011	rs9271366	*MHC-DRB1*	3e-33
**IgE levels**	6,819 European ancestry individuals	7,809 European ancestry indivduals	22075330	rs1059513	*STAT6*	2e-12
	6,819 European ancestry individuals	7,809 European ancestry indivduals	22075330	rs13962	*DARC*	2e-11
	6,819 European ancestry individuals	7,809 European ancestry indivduals	22075330	rs1801275	*IL4R*	1e-7
	1,530 European ancesrty individuals	9,769 European ancestry individuals	18846228	rs2040704	*RAD50*	4e-8
	6,819 European ancestry individuals	7,809 European ancestry indivduals	22075330	rs20541	*IL13*	3e-18
	1,530 European ancesrty individuals	9,769 European ancestry individuals	18846228	rs2251746	*FCER1A*	2e-20
	6,819 European ancestry individuals	7,809 European ancestry indivduals	22075330	rs2251746	*FCER1A*	5e-26
	2,469 African American individuals, 259 Latino individuals, 1,564 European ancestry individuals	2,961 African American individuals, 1,477 Latino individuals, 649 European ancestry individuals, 680 Hutterite individuals	23146381	rs2363709	*SUCLG2*	0.000005
	6,819 European ancestry individuals	7,809 European ancestry indivduals	22075330	rs2523809	*MHC-G*	4e-8
	6,819 European ancestry individuals	7,809 European ancestry indivduals	22075330	rs2571391	*MHC-A*	1e-15
	6,819 European ancestry individuals	7,809 European ancestry indivduals	22075330	rs2858331	*MHC-DQA2*	1e-8
	1,854 Hispanic asthmatic individuals, 1,480 Hispanic individuals	454 Hispanic asthmatic individuals	25488688	rs3024667	*IL4R*	0.000002
	6,819 European ancestry individuals	7,809 European ancestry indivduals	22075330	rs3102947	*ID2*	2e-7
	967 Japanese ancestry individuals, 213 Japanese ancestry asthmatic individuals	1,894 Japanese ancestry individuals, 580 Japanese ancestry asthmatic individuals	24324648	rs3130941	*HCG27*	1e-10
	6,819 European ancestry individuals	7,809 European ancestry indivduals	22075330	rs4656784	*OR10J3*	2e-16
	2,469 African American individuals, 259 Latino individuals, 1,564 European ancestry individuals	2,961 African American individuals, 1,477 Latino individuals, 649 European ancestry individuals, 680 Hutterite individuals	23146381	rs6499255	*WWP2*	0.000001
	6,819 European ancestry individuals	7,809 European ancestry indivduals	22075330	rs9290877	*LPP*	0.000002
	2,469 African American individuals, 259 Latino individuals, 1,564 European ancestry individuals	2,961 African American individuals, 1,477 Latino individuals, 649 European ancestry individuals, 680 Hutterite individuals	23146381	rs9469220	*MHC-DQB1*	2e-7
**IgG levels**	229 European ancestry multiple sclerosis cases	409 European ancestry multiple sclerosis cases	23225573	rs10136766	*IGHG1*	8e-16

Table S26- MR estimates from each method of the causal effect of the exposures (variants of current studies and GWAS studies) on infectious diseases as an outcome

Exposure	Method Wald ratio	Lower respiratory infection UKB-a:540	Staphylococcal infection UKB-b:3266	Bacterial infection UKB-b:1605	Other bacterial infections UKB-b:1399	Streptococcus infection UKB-b:4251	Streptococcal infection UKB-b:4884
**IgG**	**β**	-0.0001413	-0.00005549	-0.00003081	0.00003191	-0.00003168	-0.0001744
	***P*-value**	0.8738	0.3831	0.637	0.616	0.531	0.002811*
**IgA**	**β**	-0.0009505	0.0001028	-0.0002886	0.0001031	0.00001118	-0.00008361
	***P*-value**	0.6219	0.4543	0.04062*	0.4529	0.9185	0.5072
**IgE**	**β**	0.00001112	-0.000001189	0.000001105	-0.000002169	-2.856e-7	8.597e-7
	***P*-value**	0.5133	0.3255	0.3731	0.07283	0.7663	0.4384

Table S27- Variability in the causal estimates obtained for each SNP

Exposure	Outcome	method	Q	Q_df	Q_pval
**IgE**	**lower respiratory infection**	MR Egger	0.5237	5	0.9912
		Inverse variance weighted	0.6141	6	0.9962
	**Staphylococcal infection**	MR Egger	6.416	5	0.2678
		Inverse variance weighted	10.44	6	0.1074
	**Bacterial infection**	MR Egger	2.527	5	0.7724
		Inverse variance weighted	3.187	6	0.7851
	**Other bacterial infections**	MR Egger	0.646	5	0.9858
		Inverse variance weighted	0.7814	6	0.9926
	**Streptococcus infection**	MR Egger	2.857	5	0.722
		Inverse variance weighted	3.373	6	0.7608
	**Streptococcal infection**	MR Egger	1.8	5	0.8761
		Inverse variance weighted	1.809	6	0.9364
**IgA**	**lower respiratory infection**	MR Egger	18.76	7	0.008979*
		Inverse variance weighted	18.76	8	0.0162*
	**Staphylococcal infection**	MR Egger	7.238	8	0.5112
		Inverse variance weighted	7.249	9	0.6112
	**Bacterial infection**	MR Egger	2.053	8	0.9794
		Inverse variance weighted	3.643	9	0.9333
	**Other bacterial infections**	MR Egger	9.068	8	0.3366
		Inverse variance weighted	9.084	9	0.4295
	**Streptococcus infection**	MR Egger	9.796	8	0.2796
		Inverse variance weighted	9.849	9	0.3628
	**Streptococcal infection**	MR Egger	8.932	8	0.348
		Inverse variance weighted	9.54	9	0.389

Table S28- MR estimates from each method of the causal effect of the exposures (variants of current studies and GWAS studies) on the infectious diseases as an outcome

Outomes	MR method	Q	Q_df	Q_pval
**Lower respiratory infection**	MR Egger	7.588	7	0.3704
	Inverse variance weighted	8.548	8	0.3819
**Staphylococcal infection**	MR Egger	14.02	7	0.05088
	Inverse variance weighted	14.03	8	0.08103
**Bacterial infection**	MR Egger	3.801	7	0.8024
	Inverse variance weighted	5.621	8	0.6896
**Other bacterial infections**	MR Egger	3.342	7	0.8517
	Inverse variance weighted	3.452	8	0.9029
**Streptococcus infection**	MR Egger	1.79	7	0.124
	Inverse variance weighted	1.79	8	0.2191
**Streptococcal infection**	MR Egger	5.79	7	0.5645
	Inverse variance weighted	6.327	8	0.6106

Table S29- Top ten signaling pathways significantly enriched by having SNPs included in MR estimates.

Pathway identifier	Pathway name	#Entities found	#Entities total	Entities ratio	Entities pValue	Entities FDR	#Reactions found	#Reactions total	Reactions ratio	Species name
R-HSA-3906995	Diseases associated with O-glycosylation of proteins	5	76	0.005406	0.001745	0.113397	4	9	7.51E-04	Homo sapiens
R-HSA-5357786	TNFR1-induced proapoptotic signaling	2	14	9.96E-04	0.010963	0.427553	2	3	2.50E-04	Homo sapiens
R-HSA-5173105	O-linked glycosylation	5	132	0.009389	0.016848	0.582499	19	26	0.002169559	Homo sapiens
R-HSA-5678420	Defective ABCC9 causes dilated cardiomyopathy 10, familial atrial fibrillation 12 and hypertrichotic osteochondrodysplasia	1	2	1.42E-04	0.022088	0.582499	1	1	8.34E-05	Homo sapiens
R-HSA-3781865	Diseases of glycosylation	6	194	0.013799	0.022455	0.582499	8	77	0.006425234	Homo sapiens
R-HSA-174824	Plasma lipoprotein assembly, remodeling, and clearance	4	98	0.006971	0.024843	0.582499	9	84	0.007009346	Homo sapiens
R-HSA-5621481	C-type lectin receptors (CLRs)	6	202	0.014368	0.026651	0.582499	5	68	0.005674232	Homo sapiens
R-HSA-8964038	LDL clearance	2	28	0.001992	0.039639	0.582499	4	19	0.001585447	Homo sapiens
R-HSA-1855167	Synthesis of pyrophosphates in the cytosol	2	29	0.002063	0.042218	0.582499	4	15	0.001251669	Homo sapiens
R-HSA-4793950	Defective MAN1B1 causes MRT15	1	4	2.85E-04	0.043692	0.582499	4	4	3.34E-04	Homo sapiens

Figure S1- Forest plots depicting causal effect of every single significant SNP associated with IgA leves in GWAS studies predicting diverse outcomes for infectious diseases including (**A**) lower respiratory tract infections (**B**) staphylococcal infections, (**C**) bacterial infections, (**D**) unspecific bacterial infections, (**E**) streptococcus infections, (**F**) and unspecific streptococcal infections.

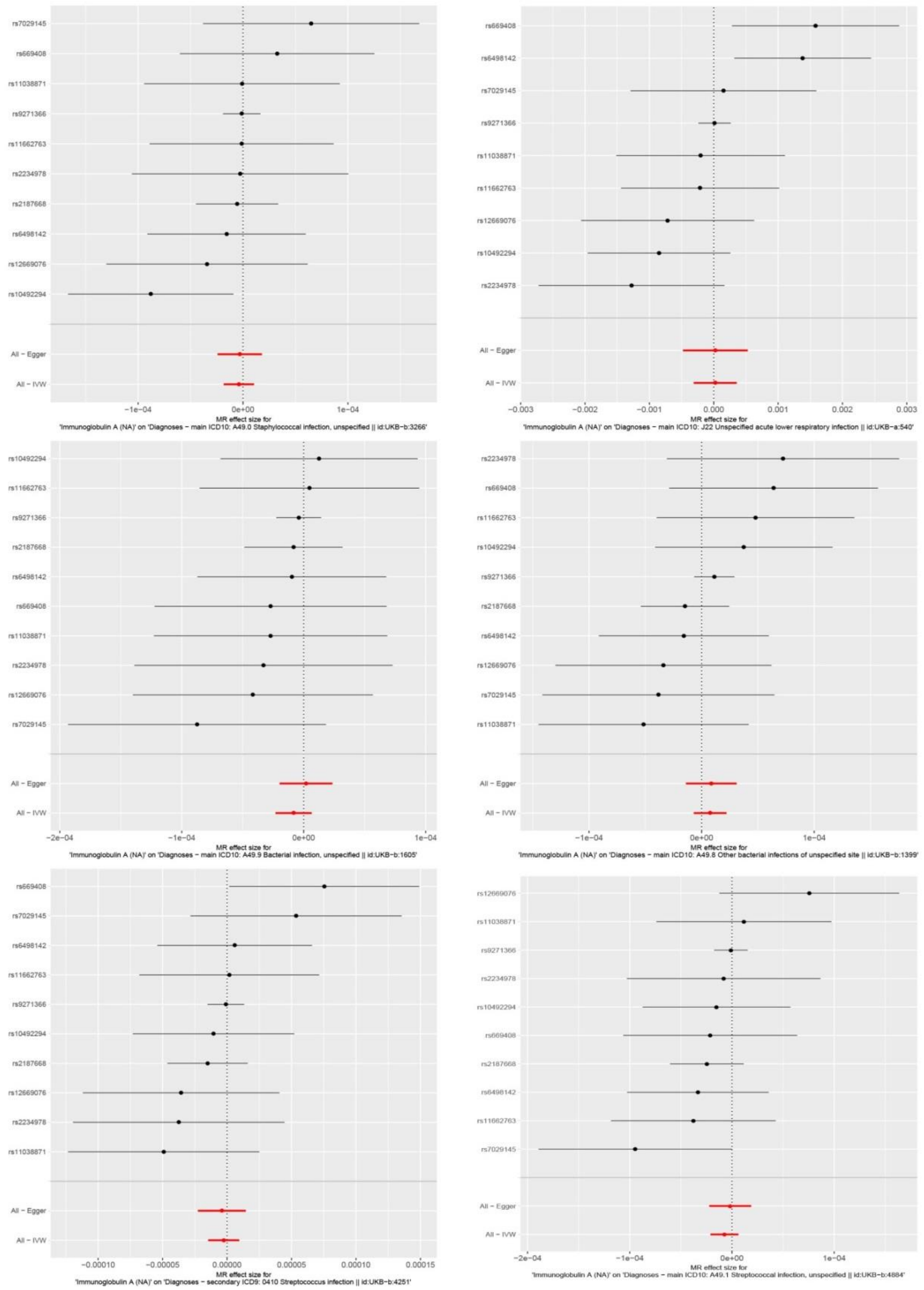

Figure S2- Forest plots depicting the causal effect of every single significant SNP associated with IgE levels in GWAS studies predicting diversely outcomes for infectious diseases including (**A**) lower respiratory tract infections (**B**) staphylococcal infections, (**C**) bacterial infections, (**D**) unspecific bacterial infections, (**E**) streptococcus infections, (**F**) and unspecific streptococcal infections.

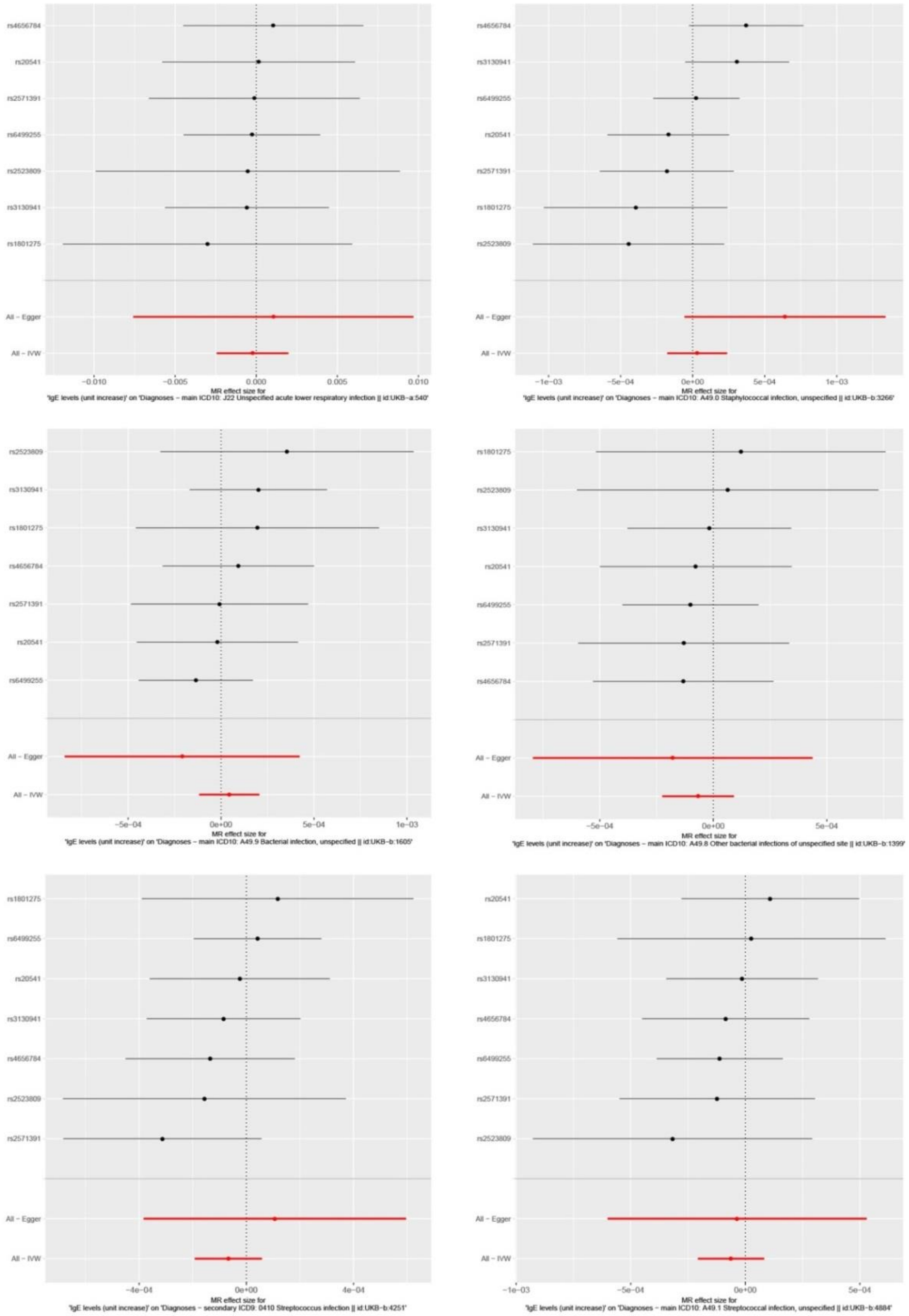

Figure S3- Funnal plots predicting the heterogeneity and presence of horizontal pleiotropy of SNP associated with IgA leves in GWAS studieson outcomes for infectious diseases including (**A**) lower respiratory tract infections (**B**) staphylococcal infections, (**C**) bacterial infections, (**D**) unspecific bacterial infections, (**E**) streptococcus infections, (**F**) and unspecific streptococcal infections.

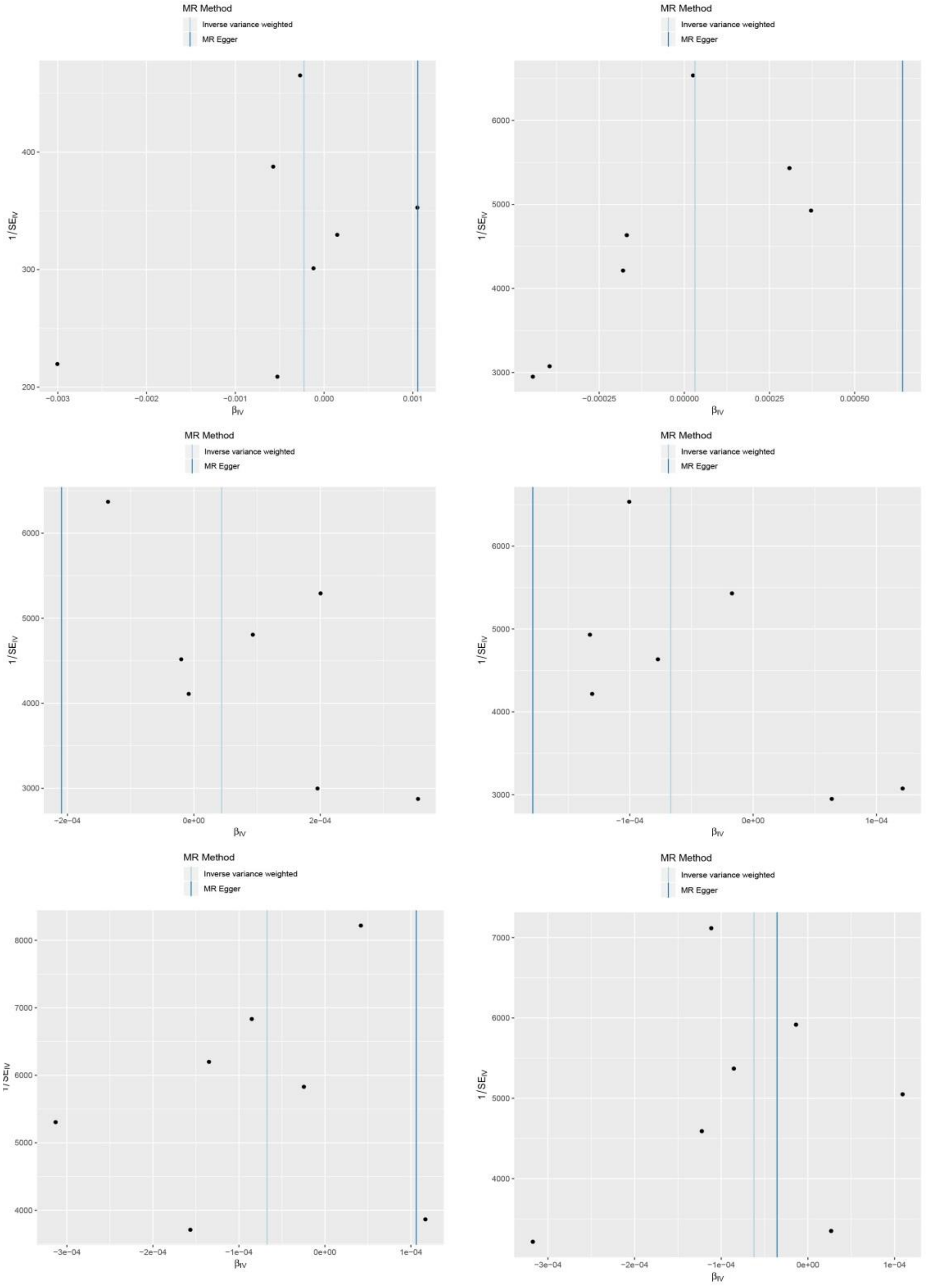

Figure S4- Funnal plots predicting the heterogeneity and presence of horizontal pleiotropy of SNP associated with IgE levels in GWAS studies on outcomes for infectious diseases including (**A**) lower respiratory infections (**B**) staphylococcal infections, (**C**) bacterial infections, (**D**) unspecific bacterial infections, (**E**) streptococcus infections, (**F**) and unspecific streptococcal infections.

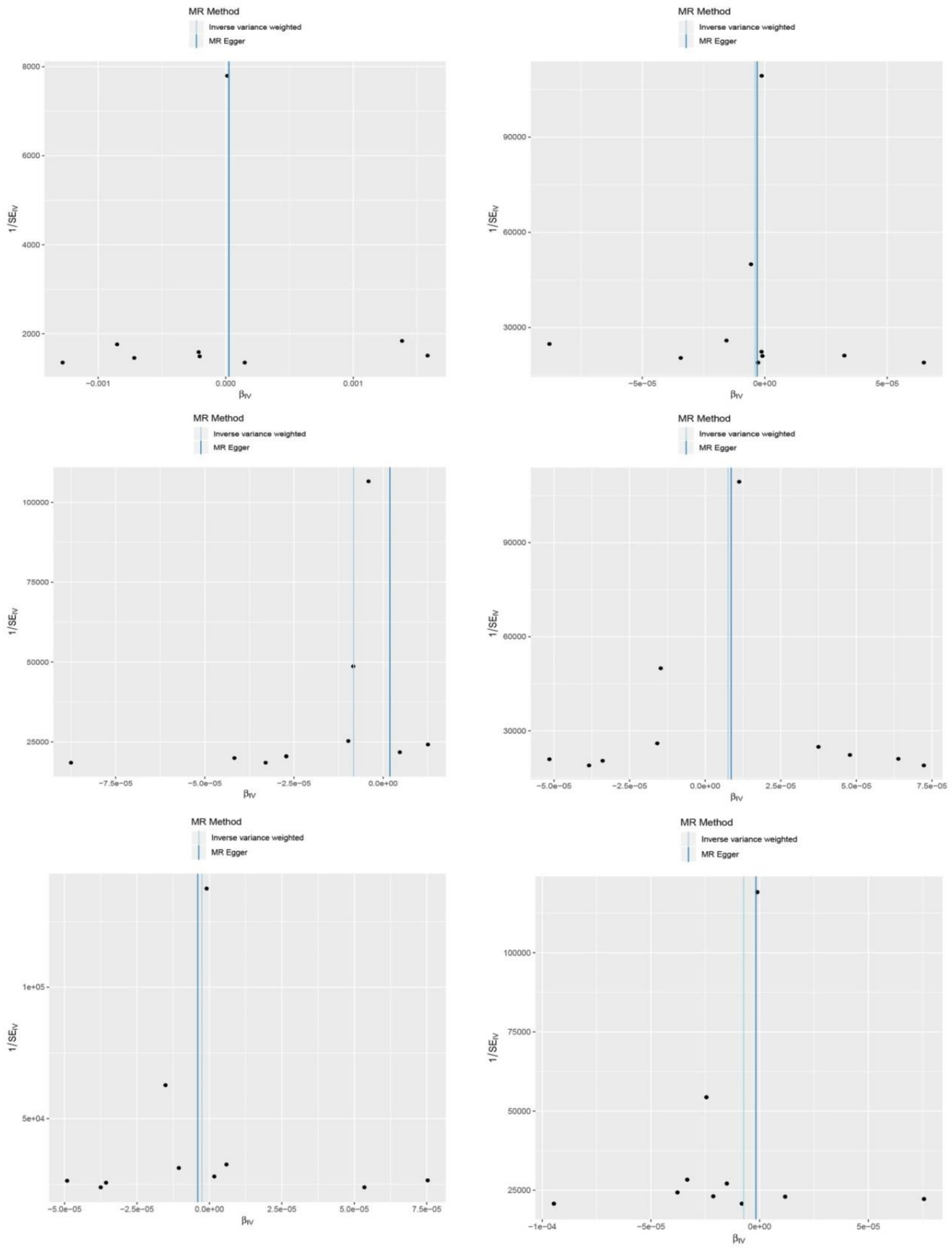

Figure S5- Funnal plots predicting the heterogeneity and presence of horizontal pleiotropy of SNP associated with unsolved CVID and outcomes for infectious diseases including (**A**) lower respiratory tract infections (**B**) staphylococcal infections, (**C**) bacterial infections, (**D**) unspecific bacterial infections, (**E**) streptococcus infections, **(F)** and unspecific streptococcal infections.

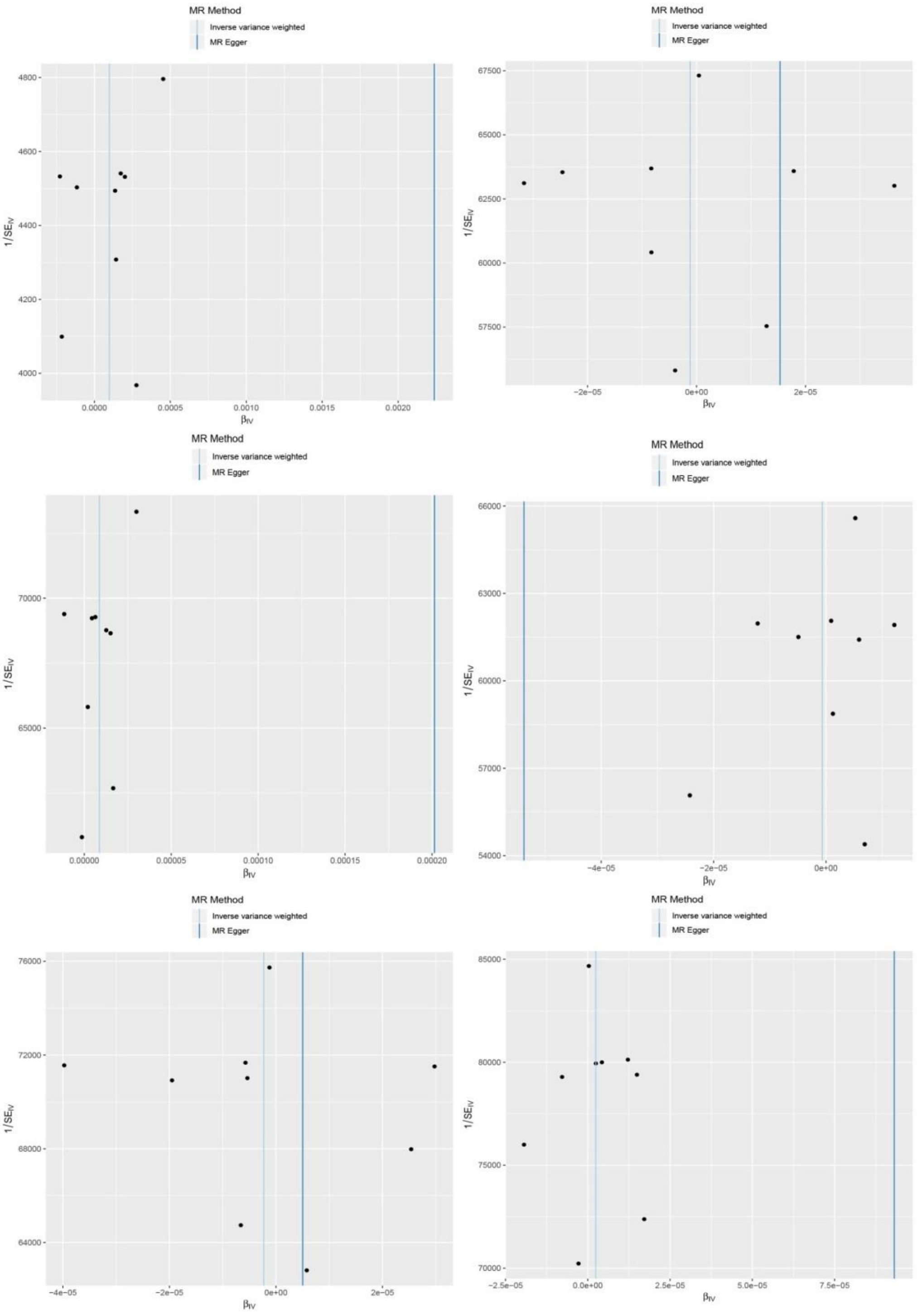

Supplement: Supplementary file 1 [file Data_Sheet_1.pdf]
